# Barcoded overexpression screens in gut Bacteroidales identify genes with roles in carbon utilization and stress resistance

**DOI:** 10.1038/s41467-024-50124-3

**Published:** 2024-08-05

**Authors:** Yolanda Y. Huang, Morgan N. Price, Allison Hung, Omree Gal-Oz, Surya Tripathi, Christopher W. Smith, Davian Ho, Héloïse Carion, Adam M. Deutschbauer, Adam P. Arkin

**Affiliations:** 1 Environmental Genomics and Systems Biology Division, Lawrence Berkeley National Laboratory, Berkeley, CA USA; 2 Department of Microbiology and Immunology, University at Buffalo, State University of New York, Buffalo, NY USA; 3 Department of Molecular and Cell Biology, University of California-Berkeley, Berkeley, CA USA; 4 Department of Plant and Microbial Biology, University of California-Berkeley, Berkeley, CA USA; 5 Department of Bioengineering, University of California-Berkeley, Berkeley, CA USA

**Keywords:** Bacteriology, Bacterial systems biology, Functional genomics

## Abstract

A mechanistic understanding of host-microbe interactions in the gut microbiome is hindered by poorly annotated bacterial genomes. While functional genomics can generate large gene-to-phenotype datasets to accelerate functional discovery, their applications to study gut anaerobes have been limited. For instance, most gain-of-function screens of gut-derived genes have been performed in *Escherichia coli* and assayed in a small number of conditions. To address these challenges, we develop Barcoded Overexpression BActerial shotgun library sequencing (Boba-seq). We demonstrate the power of this approach by assaying genes from diverse gut Bacteroidales overexpressed in *Bacteroides thetaiotaomicron*. From hundreds of experiments, we identify new functions and phenotypes for 29 genes important for carbohydrate metabolism or tolerance to antibiotics or bile salts. Highlights include the discovery of a d-glucosamine kinase, a raffinose transporter, and several routes that increase tolerance to ceftriaxone and bile salts through lipid biosynthesis. This approach can be readily applied to develop screens in other strains and additional phenotypic assays.

## Abstract (web summary)

Here, the authors developed a novel high-throughput, DNA barcoded overexpression platform, BOBA-seq, to uncover new gene functions among gut anaerobes involved in carbon utilization and stress tolerance.

## Introduction

The past decade has seen massive sequencing initiatives and -omics studies on the human gut microbiome^1–4^. We now appreciate that there is tremendous complexity in the network of interactions between host and microbes, and among microbes in this ecosystem. Despite their importance for human health, we lack a detailed mechanistic understanding of microbial functions in the gut^5,6^. The vast majority of bacterial genes have not been experimentally characterized, which in combination with their high functional diversity, has led to a knowledge gap in the field^7,8^. Accurately predicting gene functions often requires both a mechanistic understanding of the protein family and supporting phenotypic data, which can be difficult to obtain.

Functional genomic libraries can rapidly connect genotypes to phenotypes. Moreover, DNA barcoding of individual strains allows for parallel analysis of strain fitness in genome-wide libraries through barcode sequencing (BarSeq)^9^. In recent years, randomly barcoded transposon sequencing (RB-TnSeq) mutant libraries have enabled high-throughput genetic screens across hundreds of conditions in a cost-effective manner and have accelerated functional assignments of genes in diverse bacteria, including the human gut anaerobe *Bacteroides thetaiotaomicron* (*B. theta*)^10–12^. Gain-of-function screens are complementary to loss-of-function screens and have the advantage of capturing genes from bacteria that have yet to be cultivated or genetically modified. However, most libraries expressing gut-derived DNA have relied on *E. coli* as the overexpression strain, which is phylogenetically divergent from members of the abundant gut phyla Bacteroidota (formerly Bacteroidetes) and Bacillota (formerly Firmicutes)^13–18^. In particular, promoter recognition of *Bacteroides* genes in *E. coli* can be limiting and *Bacteroides* encode unique ribosomal binding sites (RBSs), which could present barriers for protein expression in *E. coli*^19–21^. Therefore, Bacteroidales serve as more suitable overexpression platforms to study functions in the gut, yet few genetic screens have been performed in them^22,23^.

Another challenge in gain-of-function screens is the reliance on deep sequencing or isolation of individual strains to identify beneficial genes^13–18,22^. This is time-consuming and laborious to perform for hundreds of assays, which has limited these approaches to a small number of conditions. Random DNA barcoding of libraries can drastically increase the scale of overexpression screens. For example, dual barcoding of vectors (Dub-seq) has been used to construct libraries expressed in *E. coli*, but these are currently limited to ~250,000 strains and are restricted to DNA sources with genome assemblies, which are required for mapping^24–27^.

Here, we exploit advances in long-read sequencing to develop a new workflow termed Boba-seq, or Barcoded Overexpression BActerial shotgun library sequencing, to perform large-scale gain-of-function screens using DNA barcoded vector libraries. We demonstrate this with overexpression in *B. theta*, although this approach is readily applicable to alternative expression strains and is agnostic to the source of DNA being assayed. Bacteroidales are major constituents of the gut microbiota that establish stable, long-term associations with the human host. They play crucial ecological roles in the gut, including metabolism of diverse, host-recalcitrant carbohydrates and modulation of the human immune system^28^.

We constructed Boba-seq libraries from seven Bacteroidales and first validated our method in a complementation screen. We then assayed a pool of six libraries in competitive fitness assays and identified genes involved in the metabolism of 15 carbon substrates and tolerance to seven inhibitory compounds (antibiotics or bile salts). Genes with novel functions or phenotypes included enzymes, transporters, and regulators. In addition to gene function discovery, our work enables large-scale gain-of-function screens in a clade of gut bacteria important for human health.

## Results

### Overview of workflow to build and assay barcoded overexpression libraries

**Fig. 1:**
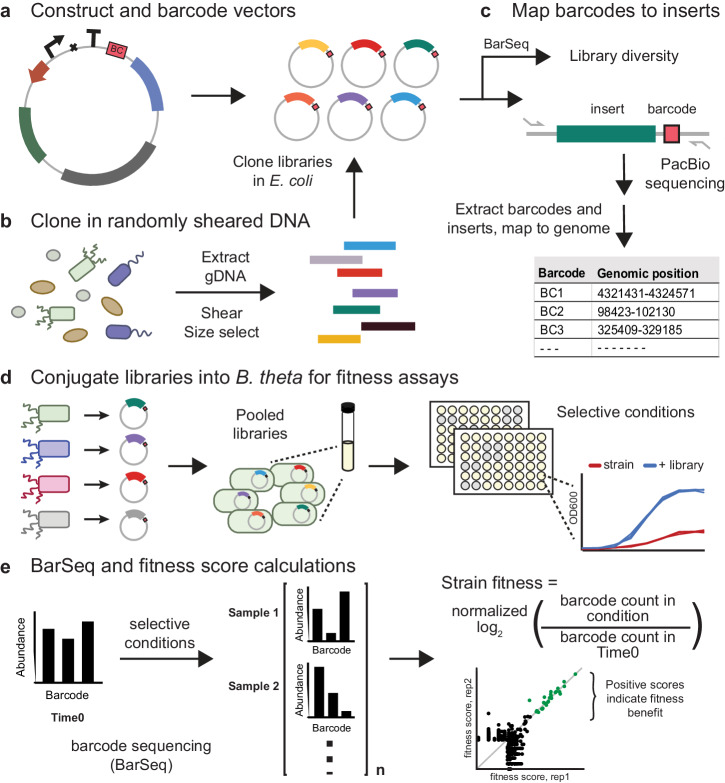
The Boba-seq workflow for high-throughput functional screens. **a** Clone DNA barcodes into vectors that replicate in the final overexpression host. **b** Extract DNA, shear, size-select, and insert into barcoded vectors using an *E. coli* cloning strain. **c** Quantify library diversity using BarSeq. Map barcodes to inserts using long-read sequencing technology and generate a barcode-to-insert mapping table. **d** Conjugate mapped libraries into the overexpression strain for fitness screens. Libraries can be pooled to increase the genetic diversity assayed. A range of selective conditions can be used to determine fitness differences across strains, such as carbon utilization and stress tolerance. **e** Quantify library composition before and after growth on selective conditions using BarSeq. A fitness score is calculated for each barcode or strain. Significantly high fitness scores in both replicates point to putative beneficial genes in the condition.

The Boba-seq workflow utilizes a single DNA barcode per plasmid to increase the throughput of phenotypic assays. This approach consists of five general steps (Fig. 1). First, we construct replicative vectors to encode an inducible promoter, an insertion site for DNA of interest, a terminator, a DNA barcoding site, and an origin of replication for the overexpression host. Empty vectors are barcoded by cloning in random 20 nucleotide barcodes^29^. In the second step, randomly sheared and size-selected DNA fragments from the organism(s) of interest are cloned into the barcoded vectors. Here, fragment sizes can be optimized to capture single genes or larger gene clusters that might encode the target phenotypes. Next, fragments are linked to unique barcodes using long-read sequencing. This simplifies the mapping step to a single PCR amplification step instead of the complex TnSeq-like protocol that was used previously to map dual-barcoded vectors^24^. In step four, libraries are conjugated into the overexpression strain. In the final step, libraries are screened across a panel of conditions where the inoculum (Time0) and samples grown in selective media are harvested for BarSeq. Inserts that lead to increased strain fitness are identified by calculating fitness scores for each barcode, which represents the log2 change in the relative abundance of the strain. Fitness scores are then filtered to identify potential causative genes for the observed fitness gain.

### Construction of DNA barcoded overexpression libraries from Bacteroidales genomic DNA

**Fig. 2:**
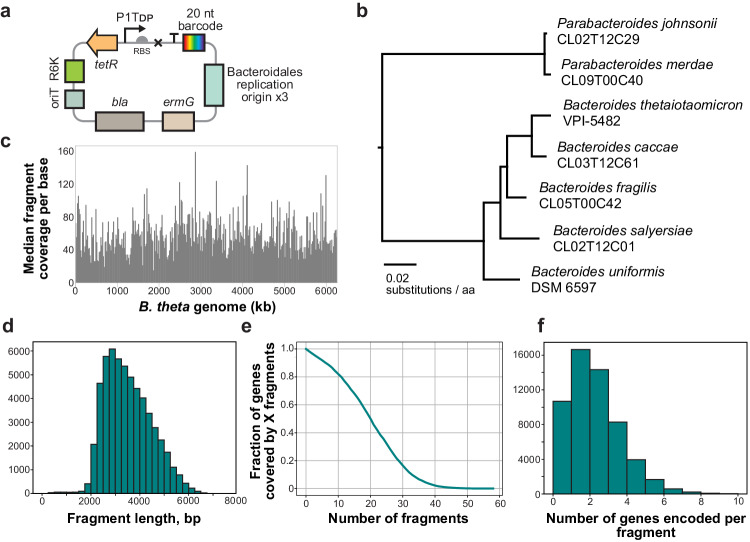
Boba-seq libraries were constructed from gDNA of *Bacteroides* and *Parabacteroides* isolates with high genomic coverage. **a** Three replicative vectors were constructed by modifying an integrative vector (pNBU2-ErmG)^33^. **b** 24 universal bacterial marker genes from the Genome Taxonomy Database (GTDB) were used to generate this phylogenetic tree to show phylogenetic relatedness of the seven isolates used to build Boba-seq libraries^88^. Protein sequences of marker genes were aligned with HMMer 3.3.1 (hmmer.org) and then a tree was inferred with FastTree 2.1.11^89^ and midpoint rooted. Coverage of the *B. theta* genomic library: **c** fragment coverage per base across the genome (NC_004663.1) plotted in 20 kb windows, (**d**) histogram of fragment lengths, (**e**) cumulative gene coverage plot, and (**f**) histogram of number of full-length genes encoded per fragment with 0 as the first bin.

We generated and characterized new shuttle vectors for *B. theta* VPI-5482 (see Methods)^30–33^. This led to three replicative vectors: pNBU2_repA1, pNBU2_repA2, and pNBU2_repA3 (Fig. 2a, Supplementary Data 1). Our vectors contain an anhydrotetracycline (aTc) inducible promoter, a RBS, and a terminator^33–35^. Inducible gene expression by aTc was verified using NanoLuc reporter protein (Supplementary Fig. 1a). The plasmid copy number for these vectors in *B. theta* was determined to be around 3, 7, and 8, respectively, using droplet digital PCR (Supplementary Fig. 1c). Collectively, these vectors are replicative in 10 of the 13 Bacteroidales tested, including *B. theta* (Supplementary Fig. 1d). The vector pNBU2_repA1 was then barcoded to contain approximately 14 million unique barcodes (see Methods). Insert lengths of ~3 kb were selected to capture at least one gene on most fragments. In total, seven libraries were generated using gDNA from *B. theta*, *B. caccae*, *B. fragilis*, *B. salyersiae*, *B. uniformis*, *Parabacteroides johnsonii*, and *Parabacteroides merdae*, which share a maximum of 92% average nucleotide identity. Phylogenetic relationships between these seven strains are shown in Fig. 2b. 81% of all protein-coding genes in these genomes have at least one protein homolog (≥40% a.a. identity, ≥75% coverage), either in the same genome or in another genome.

Next, we developed a pipeline to link barcodes to fragments on each vector (Supplementary Fig. 2). We performed PacBio amplicon sequencing of the insert-barcode region to obtain 10–24-fold sequencing depths (Supplementary Data 2). 77–91% of barcodes detected by BarSeq were mapped, hence only a small fraction of each library is not analyzed. On a per genome basis, fragments cover 87–98% of protein-coding genes with 76–477 protein-coding genes unmapped (Supplementary Data 2, Fig. 2c). In all libraries, the missing proteins are generally longer than mapped proteins (median 1758 vs. 927 bp) and 5% have a nearly-identical copy (≥95% a.a. identity) elsewhere in the genome. Overall, 50% of the missing proteins are over 2 kb in length or are duplicated, which reflects limitations imposed by fragment sizes and difficulties in mapping highly similar regions. After excluding genes of over 1.5 kb or with duplicates, 31% of the remaining genes have a homolog in the same or another library that is also missing (Supplementary Data 3). Conserved missing genes suggest possible toxicity in *E. coli* and include proteins that are resistant to horizontal gene transfer such as ribosomal subunits, although the majority cannot be readily explained based on the annotation^36^.

Libraries consist of barcode diversities ranging from 40,911 to 97,471 and median fragment lengths of 2.6–3.7 kb (Fig. 2d, Supplementary Fig. 3a, Supplementary Data 2). The majority of fragments encode 1–2 complete genes (regardless of orientation) and the median fragment coverage per gene is 12–24 (Fig. 2e, f, Supplementary Fig. 3b, c, Supplementary Data 2). Overall, 50,000 fragments of ~3.1 kb are sufficient to cover a bacterial genome of approximately 5–6.5 Mbp with a median fragment coverage of >10. A sequencing depth of >10× with long reads can map a majority of barcodes. In summary, we constructed seven libraries with high genome-wide coverages.

### Complementation assays as a proof-of-concept

To validate our workflow in a phenotypic screen, we complemented *B. theta* ΔBT2158 and ΔBT3703 with *B. theta, B. fragilis, B. uniformis*, and *P. johnsonii* libraries^11^. BT2158 (BT_RS10925) encodes a periplasmic glycoside 3-dehydrogenase required for disaccharide and glucosinolate metabolism^11,37^. ΔBT2158 does not grow on trehalose (glucose-α-1,1-glucose), leucrose (glucose-α-1,5-fructose), or palatinose (glucose-α-1,6-fructose) (Supplementary Fig. 4). The second mutant lacks the ɑ-SusB (BT3703/BT_RS18670) part of the starch utilization system and has reduced growth on ɑ-cyclodextrin compared to wild-type^11^. We then inoculated each library in a defined minimal medium (Varel-Bryant or VB) containing a single carbon substrate in the presence of the inducer aTc (Supplementary Data 4)^38^. Among the four source genomes, three encode BT2158 or a homolog, and three encode BT3703 or a homolog, which are expected to provide fitness gain in the conditions tested.

We computed fitness scores for each fragment using the log2 fold-change of each barcode’s relative abundance between the selective condition and the Time0. We focus on high positive scores because Boba-seq is a gain-of-function assay and we established filters to identify biologically consistent hits. First, fragments with a fitness score ≥5 and a z score ≥4 in both replicates are considered statistically significant. Using these thresholds, no significant scores were found in a control comparison of Time0 samples (see Methods). Second, we consider each hit to be biologically consistent if it is confirmed by overlap or by protein similarity. Specifically, a hit is biologically consistent if (1) there is an overlapping fragment with significant high scores or (2) there is a protein homolog (≥40% identity) from a different genomic region (within the same library or a different library) with a significant fitness score. Finally, we inspect the gene(s) covered by these fragments to identify the causative gene. This led to 16 biologically consistent region by condition pairs encoding 15 unique proteins that conferred a fitness gain in leucrose, palatinose, or ɑ-cyclodextrin (Supplementary Data 5).

**Fig. 3:**
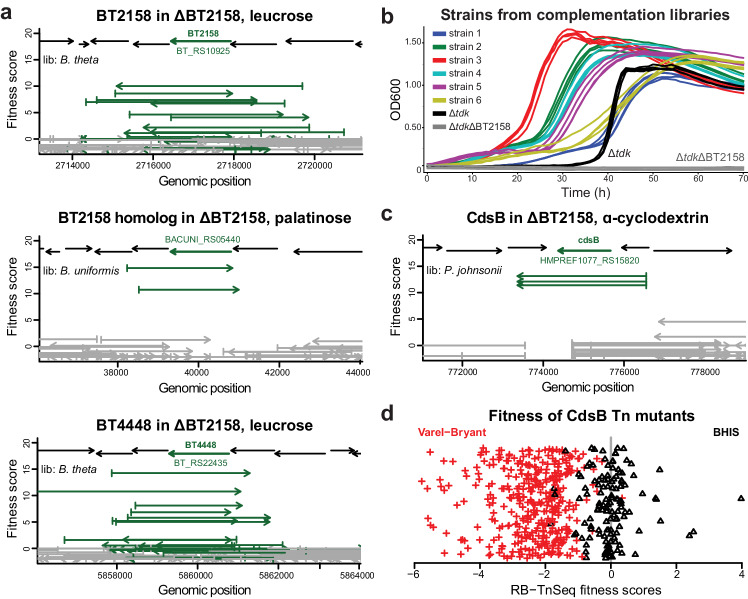
Fitness data from complementation assays. The average fitness score from both replicates is plotted for each fragment. Fragments that cover the complete gene are indicated in green. **a** BT2158 and BT4448 in *B. theta*, and a homolog (BACUNI_RS05440), were beneficial for the ΔBT2158 mutant during growth in leucrose and palatinose. **b** Growth curves of *B. theta* Δ*tdk*ΔBT2158 complemented with either BT2158 or a homologous hit. Complemented isolates, the Δ*tdk* parental strain, and the Δ*tdk*ΔBT2158 mutant were cultured in the VB medium with 20 mM trehalose. Strains 1–2 encode BT2158, strains 3–4 encode BT4448, and strains 5–6 encode BACUNI_RS05440. See Supplementary Data 7 for the precise genomic boundaries of cloned fragments. Each strain was grown in replicates of 4. OD600 values are pathlength-corrected and blank-normalized. **c** CdsB was enriched in the Δ*tdk*ΔBT3703 mutant in ɑ-cyclodextrin. **d** RB-TnSeq fitness scores for CdsB (BT2080/BT_RS10540) are averages across all 37 CdsB transposon mutants in the *B. theta* library and are log2 fold-changes that were previously reported (x-axis)^11^. Scores are highlighted in red for assays in the VB minimal medium and in black for the BHIS rich medium. The y-axis is random scatter for visualization.

For libraries expressed in ΔBT2158, growth in leucrose or palatinose led to the expected hits encoding BT2158, its nearly-identical paralog BT4448 (BT_RS22435), or a homolog (BACUNI_RS05440) from *B. uniformis* (Fig. 3a). In trehalose, we observed extremely high fitness scores (>12) for strains encoding each of these genes, but each strain had high fitness in just one replicate, so they did not meet our criteria for statistical significance. These strains likely grew quickly early on in one replicate but were outcompeted by other fit strains in the second replicate. To verify our results, we isolated clones encoding either BT2158, BT4448, or BACUNI_RS05440 and observed improved growth on trehalose (Fig. 3b). Of note, three of six fragments encode genes oriented opposite to the inducible promoter with <50 bp upstream and were likely transcribed from a weak, unexpected promoter located downstream of the vector insert site (Supplementary Data 7, Supplementary Fig. 1b).

Previously, it was observed that the ΔBT2158 mutant cannot grow on these disaccharides, suggesting that BT2158 is essential for metabolism and BT4448 cannot rescue its activity^11^. In contrast, our data show that BT4448 can functionally complement ΔBT2158 when expressed from a vector. BT4448 is likely not expressed to sufficient levels endogenously in these conditions to rescue growth in ΔBT2158 and that overexpression from our vector led to higher activity.

Complemented ΔBT3703 libraries in ɑ-cyclodextrin led to a cysteine desulfidase (CdsB) and a cysteine β-lyase as beneficial hits (Fig. 3c, Supplementary Data 5). Here, the selection for cysteine degrading enzymes appears to be stronger than for BT3703 and its homologs since ΔBT3703 is capable of growth on ɑ-cyclodextrin^11^. Cysteine degradation is likely beneficial due to toxicity of the 8.25 mM cysteine in the minimal medium (Supplementary Fig. 5)^39,40^. This is also supported by RB-TnSeq data for *B. theta*, where disruption of CdsB (BT2080/BT_RS10540) led to reduced fitness across all conditions in the same minimal medium (Fig. 3d)^11^. Similarly, cysteine β-lyase mutants of *B. theta* exhibited mild fitness defects in many conditions^11^. Of note, the rich medium used for *B. theta* RB-TnSeq fitness assays also contains 8.25 mM cysteine, and thus this effect is specific to minimal media conditions (Fig. 3d)^11^. Cysteine is commonly added to Bacteroidales growth media as a reductant, but our result cautions against its use at a high concentration in minimal media.

In summary, we established a set of stringent filters to identify beneficial hits. In particular, complementation worked well for conditions with strong selective pressure where the host strain exhibits little or no growth.

### Fitness profiling of pooled Boba-seq libraries

After establishing a pipeline for fitness data analysis, we sought to map genotypes to phenotypes in conditions relevant for the gut habitat using wild-type *B. theta* as an expression host. In total, we performed 222 assays consisting of 42 single carbon sources in a defined minimal medium and 16 inhibitory compounds at 2–5 concentrations in a rich medium (Supplementary Data 4). The vast majority of assays were carried out in the presence of the inducer aTc. Carbon sources included dietary and host-derived complex polysaccharides, gelatin, mucins, and simple carbohydrates. Inhibitory compounds consisted of antibiotics and bile salts. Six libraries (excluding the *B. theta* library) were pooled into a single inoculum (Time0). Growth was observed in 29 carbon substrates and all stress conditions. 192 samples were processed for BarSeq and included two replicates per condition and two Time0 samples per fitness screen.

**Fig. 4:**
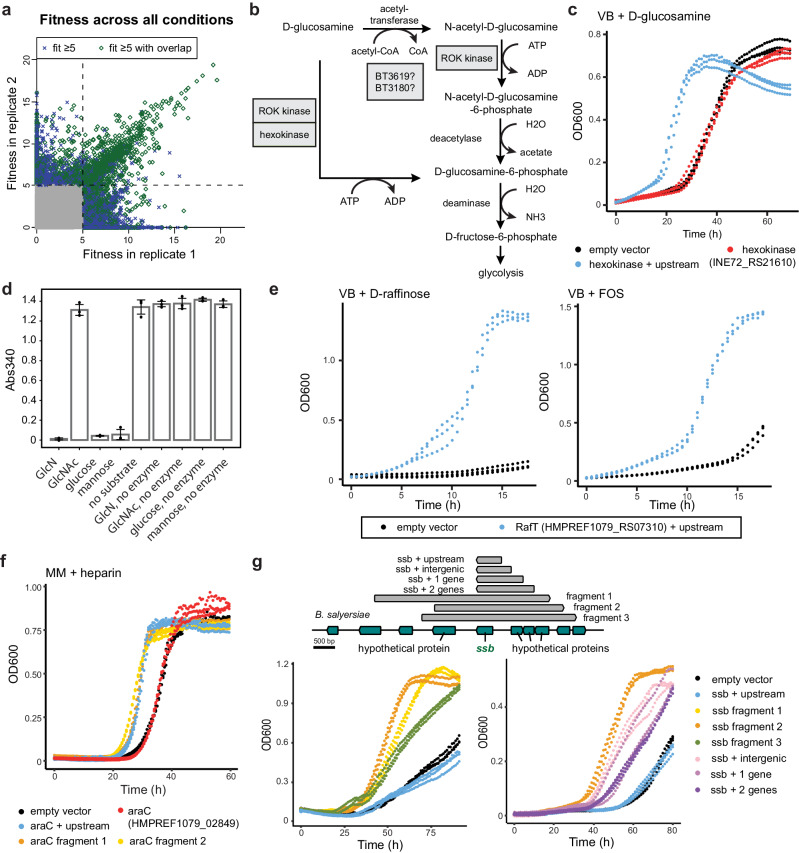
Competitive fitness assays in wild-type *B. theta* uncovered novel functions in carbon utilization. **a** Consistency of strain fitness values between two replicates across 93 pairs of experiments with 6 pooled libraries. Gray points represent fitness scores <5. Dashed lines separate fitness scores of ≥5. Barcodes with fitness scores ≥5 whose regions overlap with other high-scoring barcodes are highlighted in green. **b** d-Glucosamine and GlcNAc share a metabolic pathway in Bacteroidales. ROK kinase and hexokinase hits from our screen are displayed based on biochemical evidence. **c** A hexokinase conferred a growth advantage on d-glucosamine when overexpressed in *B. theta*. **d** In vitro end-point enzyme assays coupled to NADH depletion were performed for hexokinase to reveal a substrate preference for d-glucosamine (GlcN), glucose, and mannose. A representative experiment with replicates of three is shown. Data is shown for mean values ± standard deviation. **e** *B. theta* expressing RafT is more fit growing on raffinose and FOS as the sole carbon source. **f** An AraC regulator provides a fitness gain on heparin. **g** A genomic region containing an ssDNA-binding protein (HMPREF1071_RS19060) is beneficial on l-fucose. For all growth curves, individual strains were cloned to identify the causative region and constructs with “+ upstream” indicate the inclusion of the upstream 200 bp. The VB minimal medium was used for each condition except for heparin where a different defined minimal medium (MM)^44^ was used (Supplementary Data 4). See Supplementary Data 7 for the genomic boundaries of library fragments verified by growth assays of individual strains.

Of the 305,382 uniquely-mapped barcodes present in the six libraries in the conjugation donor strain, we detected 283,816 barcodes in our fitness assays (having at least 2 reads across all assays and controls). Across all libraries, 21,042 protein-coding genes were assayed. We identified 1172 barcodes with statistically significant scores using the same criteria as previously detailed for the complementation screen (Fig. 4a). We identified biologically consistent fragments for 120 unique regions across 22 compounds. The majority of beneficial regions were curated to assign a putative causative protein or proteins by inspecting fitness score vs. fragment coverage plots (Supplementary Fig. 6). Experimental evidence from any homolog as part of a publication was assessed using PaperBLAST and taken into consideration as well^41^. Among the curated hits, gene lengths range from 246 to 3412 bp with a median of 1281 bp (Supplementary Data 5). For comparison, the median length of a protein-coding gene across the six genomes is 957 bp. Among the highest average fitness score for each of the 200 beneficial regions, fragment sizes range from 1491 to 5243 bp with a median of 3059 bp (Supplementary Data 5). We detected beneficial genes that are absent in the *B. theta* genome as well as those with a homolog encoded by *B. theta*, demonstrating our platform can capture fitness gains caused by increased gene copies in additional to the presence of new genetic material. Overall, hits across all conditions consist of 76 protein clusters with 30 clusters containing multiple homologs at ≥40% amino acid identity (Supplementary Data 5).

We tested whether the presence of aTc may have increased the number of hits across two carbon conditions by performing the same assays with and without aTc. In fucose, we identified a greater number of hits with aTc than in its absence (64 vs. 32; *P* = 0.001, chi-squared goodness of fit test) and similarly in heparin, we observed more hits with the inducer (32 vs. 8; *P* = 0.00014). We also observed an increase in the proportion of hits containing a gene on the same strand as the inducible promoter in the presence of aTc (from 33% to 66% in fucose; from 25% to 41% in heparin). Collectively, this supports the utility of the inducible promoter for increasing gene expression and obtaining significant hits.

To lend further support to our data, we compared the 76 protein clusters that conferred enhanced fitness to loss-of-function RB-TnSeq data. We expect genes that confer enhanced fitness when overexpressed should be important for fitness when disrupted by a transposon and lead to a negative fitness score in RB-TnSeq libraries. For this analysis, we leveraged the large existing RB-TnSeq dataset for diverse bacteria, including *B. theta*^10,11^. We also generated a new RB-TnSeq mutant library in *Phocaeicola vulgatus* CL09T03C04, and profiled this library under eight of the same carbon sources used in Boba-seq assays (Supplementary Data 6). We identified 10 instances (of 76) where a protein homolog (at ≥30% identity) from an RB-TnSeq library had a significant phenotype in a matched experimental condition (Supplementary Data 5). This provides strong experimental evidence to support their importance in the corresponding biological processes, and highlights the complementarity of gain-of-function and loss-of-function genomic approaches.

Consistent with the complementation screen, we detected high fitness scores for CdsB and cysteine β-lyase across 9 conditions containing cysteine (Supplementary Data 5). In addition, 21 carbon substrates were tested using an alternative reductant, dithiothreitol (DTT). While most of these assays led to similar hits after excluding Cys-degrading enzymes, assays with DTT generally led to more hits compared to assays with cysteine.

We noticed that many strains did not show a benefit even though they encode a curated causative gene or genes (Supplementary Data 5). Specifically, 31% of strains that contain the putative causative gene(s) show a significant benefit (fitness score ≥5 and z ≥ 4 in ≥1 replicate). This is likely partly due to differences in gene expression caused by variations in fragment boundaries: for strains with the exact same insert sequence where at least one strain has a statistically significant score, 70% have a significant score in at least one replicate. This is much higher than the 31% rate for all strains with causative genes. Our vector’s inducible promoter was designed to ameliorate expression issues and aTc did increase the number of significant fitness values, but many genes are likely transcribed from their native promoters, which are not always included on fragments. Moreover, most protein-coding genes in Bacteroidales do not have an upstream Shine-Dalgarno sequence and the mechanism for translation initiation without the Shine-Dalgarno sequence is not understood^21,42^. This presents an additional variable that may influence gene expression in our libraries in ways we cannot predict.

### High-confidence hits uncover metabolic enzymes in carbohydrate utilization

Here, we focus on metabolic enzymes beneficial on single carbon sources. Three substrates led to expected hits based on known functions or annotations (Supplementary Data 5). These include the l-fucose catabolic operon from l-fucose and a β-glucosidase from d-cellobiose and d-salicin (Supplementary Fig. 6)^11,43^. We verified that the β-glucosidase confers a growth benefit when expressed in *B. theta* on d-cellobiose and d-salicin (Supplementary Fig. 7a).

Additionally, an ɑ-glucosidase (INE72_RS06280) in the glycosyl hydrolase family 31 was beneficial in palatinose in both the pooled library assays and when overexpressed in individual strains (Supplementary Figs. 6, 7b). Since palatinose contains an α-1,6-glycosidic bond, we annotate this enzyme as an α-1,6-glucosidase.

An ROK family (repressor, open reading frame, kinase) protein and a hexokinase increased fitness on d-glucosamine. We first show that strains encoding the ROK kinase or the hexokinase have a growth advantage in d-glucosamine (Fig. 4c, Supplementary Fig. 7c). In support of our data, an ROK kinase homolog in *B. theta* (31% identity) can phosphorylate d-glucosamine and N-acetyl-d-glucosamine (GlcNAc) in vitro^44^.

In *B. theta*, d-glucosamine and GlcNAc share a metabolic pathway where d-glucosamine can be acetylated to form GlcNAc (Fig. 4b)^45^. GlcNAc is then phosphorylated and deacetylated to form d-glucosamine-6-phosphate^46^. While two kinases were beneficial for growth on d-glucosamine, no hits were observed in GlcNAc, which may be a result of lower selective pressure since *B. theta* grows better on GlcNAc than d-glucosamine in the VB medium. The hexokinase is important for glucose and mannose metabolism as previously reported using a *B. fragilis* deletion mutant (AY664812, 79% identity)^47^. While our fitness data support d-glucosamine as an additional substrate, we cannot exclude the possibility of GlcNAc phosphorylation by this hexokinase since it is an intermediate of the metabolic pathway (Fig. 4b). Therefore, we purified the hexokinase (INE72_RS21610) to establish its activity in a coupled enzyme assay. The hexokinase has a strong substrate preference for glucose, mannose, and d-glucosamine, with little or no activity toward GlcNAc (Fig. 4d). Here, we use both genetics and biochemical approaches to identify a new d-glucosamine kinase and demonstrate its importance in d-glucosamine metabolism in Bacteroidales.

### TonB-dependent transporters mediate raffinose uptake

The TonB-dependent transporter (TBDT) family can carry out active transport of vitamins, siderophores, peptides, and glycans across the outer membrane, and are prevalent in the phylum Bacteroidota, with 121 putative TBDTs in *B. theta* alone^48^. Two TBDTs were uncovered to be beneficial for growth in raffinose and fructooligosaccharides (FOS) (Supplementary Data 5). To our knowledge, this is the first report of raffinose transporters in Bacteroidales. Raffinose is a trisaccharide part of the raffinose family of oligosaccharides (RFOs) that is widespread in dietary plants and abundant in the standard mouse chow used in microbiome studies^49,50^. A distantly-related TBDT from *B. theta* (BT1763/BT_RS08935, ≤28% identity) is involved in the uptake of FOS with β-2,6 linkages, but the FOS substrate used in our study contains predominantly β-2,1-linkages (Supplementary Data 4)^51^.

We name these transporters RafT as they share similarity at 49% amino acid identity (HMPREF1077_RS18310, HMPREF1079_RS07310), but have distinct gene neighborhoods. An α-galactosidase is present downstream of *Pj-*RafT (from *P. johnsonii*) and can likely release galactose from raffinose^52^. A β-d-fructofuranosidase is genomically adjacent to *Bf-*RafT (from *B. fragilis*) and can hydrolyze raffinose^53^. Both nearby enzymes are predicted to be periplasmic, which supports a direct uptake of raffinose by the RafTs. This is also supported by RB-TnSeq data for a RafT homolog in *P. vulgatus* (HMPREF1058_RS00615, >47% identity) that is important for raffinose utilization. In addition to raffinose, *Bf*-RafT was also a hit in FOS and in the complementation screen for leucrose. Leucrose, raffinose, and FOS all contain d-fructose as a terminal residue, which might mediate substrate recognition by *Bf-*RafT. We confirmed that *Bf-*RafT is beneficial for *B. theta* grown on raffinose and FOS (Fig. 4e). Collectively, the combination of data from Boba-seq libraries, RB-TnSeq libraries, gene neighborhoods, and growth experiments, support raffinose uptake as a function of these TBDTs.

Most Bacteroidales TBDTs are SusC-like and genomically co-localize with a partner lipoprotein-encoding *susD*^48^. SusDs form complexes with SusCs and are thought to be essential for substrate capture and transport by SusC-like TBDTs^48,51^. In contrast, the RafT hits lack an adjacent *susD* gene and are likely not SusD-dependent. We likely observed benefits for lone TBDTs, and not for SusCD pairs, due to shorter library fragments since the coverage of both genes would require fragments longer than ~5 kb.

### A regulator confers fitness in heparin

In addition to genes involved in metabolic and transport pathways, our results point to regulatory mechanisms as well. An AraC family transcriptional regulator (HMPREF1079_RS07695) conferred a fitness benefit on heparin in both pooled fitness assays and individual strain growth assays (Fig. 4f, Supplementary Fig. 6). This protein has been associated with resistance to DNA damaging agents, but a role in heparin utilization or more broadly, carbohydrate metabolism, had not been reported^54,55^.

### A mobile genetic element contributes to competitive growth on l-fucose

l-Fucose is a key nutrient for Bacteroidales in the gut with sources including dietary polysaccharides and host glycoproteins such as mucins. In addition to being a carbon source, l-fucose is a component of bacterial capsular polysaccharides and fucosylated glycoproteins^56^. During nutrient-limiting conditions, *B. theta* can induce fucosylation of host glycans to increase nutrient availability for resident microbes, thus altering gut microbiota composition^57,58^. Given the importance of this molecule for host-microbe interactions, we sought to identify genes that provide a competitive growth advantage on l-fucose.

Here, l-fucose catabolic genes were beneficial across all l-fucose conditions (Supplementary Data 5). Unexpectedly, a genomic region encoding an ssDNA-binding protein (SSB) was also detected among all l-fucose conditions (Supplementary Fig. 8a). Additionally, there are three hypothetical proteins upstream of *ssb* in this conserved region, which is part of a ~ 50 kb putative mobile genetic element (MGE) present in Bacteroidales (Supplementary Fig. 8b). In support of this, a conserved recombinase and resolvase are encoded nearby. First, we verified our fitness data using three high-scoring fragments and observed growth benefits on l-fucose (Fig. 4g). Then, by using truncated fragments, *ssb* and the upstream intergenic region appears sufficient for a partial fitness gain (Fig. 4g).

SSB proteins play important roles in DNA replication, recombination, and repair, and have multiple protein binding partners^59^. Additionally, they are present on plasmids and play anti-defense roles by inhibiting SOS response and by protecting transferred ssDNA from nuclease-catalyzed degradation^60^. Based on known functions of SSB, we could not readily propose a mechanism to account for the observed growth advantage on l-fucose. We also cannot exclude the possibility that the upstream intergenic region might contribute to the phenotype. Therefore, in an attempt to gain molecular insights, we performed an RNA-seq experiment using the most fit strain encoding a library fragment and a strain with an empty vector (Supplementary Fig. 8c, Supplementary Data 8, *p* < 0.05, Wald test). Transcripts for l-fucose catabolic genes are not differentially abundant between these two strains, which rules out a direct regulatory mechanism by which this region is conferring a benefit.

A partial restriction endonuclease subunit S (BT4522/BT_RS22805) was strongly upregulated in the strain expressing the *ssb* fragment (average log2 fold-change or log2FC of 5.5). This is caused by phase-variation in a Type I restriction modification system and may be catalyzed by an adjacent recombinase. Based on transcript read alignments, the partial S subunit gene recombined with an upstream S subunit gene such that it became expressed as part of the recombined, full-length S subunit (Supplementary Fig. 8d). We also observed expression differences in capsular polysaccharide (CPS) biosynthesis, with the CPS3 locus upregulated (average log2FC of 1.46) and the CPS5 locus repressed (average log2FC of −4.6). Both CPS loci are regulated by invertible promoters in addition to the upxY transcriptional anti-terminator and upxZ *trans* locus inhibitor system^61^. Consistent with its known role, we speculate that the SSB affects recombination events and altered phase-variation at these loci. However, we cannot pinpoint a direct mechanism by which these transcriptional changes might impart a benefit on l-fucose. Despite this, we uncovered a link between this conserved region within a MGE and a growth advantage on l-fucose.

### Multiple strategies confer resistance to antibiotics

The gut microbiota is under dynamic selection pressure from host-secreted factors, dietary compounds, and xenobiotics. Therefore, we examined genes important for fitness in the presence of different classes of antibiotics, bile salts, and an antioxidant (Supplementary Data 4). Seven inhibitory compounds led to significant hits with greater fitness gains typically in the highest concentrations (Supplementary Data 5).

**Fig. 5:**
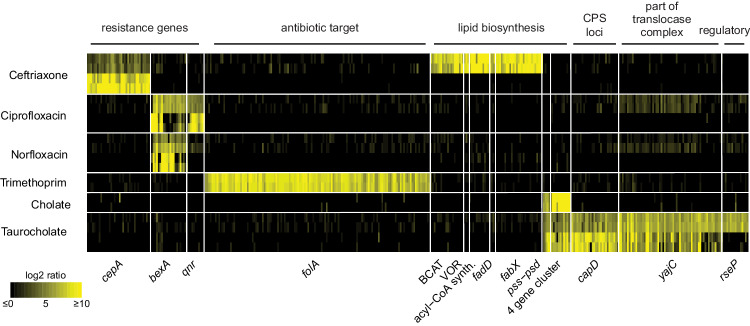
Selected gene hits that conferred benefit on antibiotics and bile salts. Fragments encoding these gene hits with significant high fitness scores from all Boba-seq libraries are displayed as fitness values (vertical lines). Replicates are plotted separately (vertically stacked) for each compound at either one or two concentrations. For compounds with hits at two concentrations, the lower concentration is plotted above the higher concentration. See Supplementary Data 5 for assay conditions, gene accessions, and proposed mechanism of tolerance. Causative gene(s) across multiple libraries are plotted within their respective protein clusters.

First, we identified three known genes (*cepA*, *bexA*, *folA*) important for antibiotic resistance through antibiotic inactivation, drug efflux, or increased target expression (Fig. 5). Additionally, a pentapeptide repeat-containing protein hit on ciprofloxacin is similar to a quinolone-resistance protein (~30% identity, EF0905) that inhibits quinolone binding to DNA gyrase and topoisomerase^62^.

**Fig. 6:**
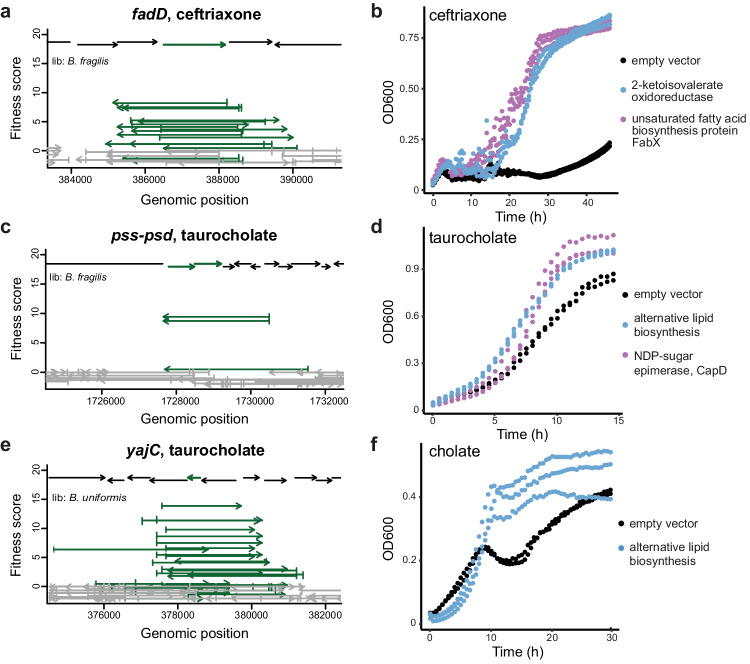
Genes involved in lipid biosynthesis, membrane protein translocation, and capsular polysaccharide biosynthesis increase tolerance to ceftriaxone, taurocholate, or cholate. Fragment plots for (**a**) *fadD* (HMPREF1079_RS19625) in 0.5 mM ceftriaxone, (**c**) phosphoethanolamine biosynthesis genes (HMPREF1079_RS14395, HMPREF1079_RS14390) in 10 mM taurocholate, and (**e**) *yajC* (BACUNI_RS18600) in 10 mM taurocholate. Growth curves of individual hits expressed in *B. theta*: (**b**) 2-ketoisovalerate oxidoreductase (HMPREF1077_RS19335, _RS17900, _RS17905, _RS17910) and *fabX* (HMPREF1077_RS10180) in 1 mM ceftriaxone; (**d**, **f**) a gene cluster for alternative lipid biosynthesis (HMPREF1077_RS03620 to HMPREF1077_RS03635) in 5 mM cholate or 5 mM taurocholate; and (**d**) an NDP-sugar epimerase *capD* (BACUNI_RS12910) in 5 mM taurocholate. All inserts include 200 bp upstream of the gene(s).

Our data also associated five genes involved in the biosynthesis of branched-chain or unsaturated lipids with ceftriaxone tolerance, a third-generation cephalosporin in clinical use (Fig. 5, Supplementary Fig. 6). Collectively, a branched-chain amino acid aminotransferase (BCAT) and a 2-ketoisovalerate oxidoreductase (VOR) catalyze the conversion of branched-chain amino acids to ɑ-ketocarboxylic acids, which are oxidatively decarboxylated to form branched-chain acyl-CoAs. These can then be converted to acyl-ACPs and undergo elongation. In support of this, the BCAT homolog in *B. fragilis* (BF9343_3671, ~88% identity) is involved in the biosynthesis of branched-chain ɑ-galactosylceramides, a type of immunomodulatory sphingolipids^63^. We identified an acyl-CoA synthetase and a long-chain fatty acid-CoA ligase (FadD) in this condition as well (Supplementary Fig. 6, Fig. 6a). Specifically, biochemical characterization of a homolog of the acyl-CoA synthetase (Q8TLW1, ~58% identity) revealed activity as a 2-methylbutyryl-CoA synthetase^64^. Finally, a beneficial nitronate monooxygenase flavoprotein was identified to be FabX as it is similar to HP0773 (~40% identity) from *Helicobacter pylori*^65,66^. FabX is essential for unsaturated fatty acyl biosynthesis and requires molecular oxygen as the electron acceptor in vitro^65,66^. However, a homolog in *Neisseria gonorrhoeae* (NGO1024, ~36% identity) is essential for anaerobic unsaturated fatty acyl biosynthesis and relies on an unknown electron acceptor^67^. We expressed BCAT, VOR, and FabX individually in *B. theta* and confirmed they are beneficial (Fig. 6b, Supplementary Fig. 7d). Our results indicate that upregulating steps in branched-chain or unsaturated fatty acyl biosynthesis increases tolerance to ceftriaxone. Changes in the outer membrane lipid composition, including sphingolipids, is a potential strategy to reduce antibiotic concentrations in the periplasm where β-lactams act. However, the precise mechanism by which this is achieved warrants further work to decipher.

### Genes in lipid and surface polysaccharide biosynthesis contribute to bile salt tolerance

Bile salts are host-secreted factors that have antibacterial activities and can modulate host signaling pathways to drive dynamic changes in the gut microbiome. These molecules act as detergents to disrupt and destabilize membrane integrity. In the colon, bacteria may encounter bile salts up to 1 mM and encode various strategies to counter their toxicity^68^. Here, we sought to identify factors that reduce susceptibility to bile stress in these microbes and thus may be important for colonization and competition in the human gut.

In both taurocholate and cholate, two beneficial loci are involved in lipid biosynthesis (Fig. 5). First, a gene cluster encoding phosphatidylserine synthase (Pss) and phosphatidylserine decarboxylase (Psd) catalyzes the formation of phosphatidylserine and phosphatidylethanolamine (Fig. 6c). These genes are essential and cannot be studied using loss-of-function approaches, highlighting an advantage of using Boba-seq. Second, a conserved cluster of four genes (adenylyltransferase/cytidyltransferase, short-chain alcohol dehydrogenase/reductase, CDP-alcohol phosphatidyltransferase, lysophospholipid acyltransferase) is absent in *B. theta* and likely involved in the biosynthesis of an alternative lipid (Supplementary Fig. 6). This operon was enriched from the two *Parabacteroides* libraries and is prevalent among *Parabacteroides*, but not *Bacteroides*. We further confirmed its benefit for growth in taurocholate and cholate (Fig. 6d, f).

In the presence of taurocholate, overexpression of RIP metallopeptidase RseP led to high fitness (Fig. 5, Supplementary Fig. 6). This is likely mediated through sigmaE factor activation by RseP in response to extracytoplasmic stress to induce transcriptional changes in cell surface proteins^69^. Additional hits on taurocholate include the preprotein translocase subunit YajC and an NDP-sugar epimerase CapD (Figs. 5, 6e). YajC is a small inner membrane protein part of the translocase protein complex with SecDF, but it is not essential and its role is not well understood^70^. In support of a role in responding to bile salts, protein levels of YajC in *Campylobacter jejuni* were elevated during exposure to chenodeoxycholate, a bile salt^71^. NDP-sugar epimerase is often part of CPS loci in *B. theta*^72^. The CPS1 locus in *B. theta* was induced in a rich medium by taurocholate but not by cholate^73^ and encodes an NDP-sugar epimerase (BT0381, >60% identity) similar to hits in our screen. Here, we further demonstrate that overexpressing the epimerase alone in *B. theta* is sufficient to increase tolerance to bile stress (Fig. 6d). Overall, both hits are supported by published gene expression data and might induce changes in membrane proteins or surface polysaccharides as a strategy to alleviate taurocholate toxicity.

In summary, our results connect genes involved in lipid biosynthesis, a regulatory pathway, membrane protein translocation, and surface polysaccharide biosynthesis to bile salt tolerance. While a few hits have supporting gene expression data from other studies, most genes are newly associated with this phenotype. Additionally, most studies quantify differentially abundant transcripts or proteins to uncover pathways involved in response to bile stress^71,73,74^. Here, we leveraged high-throughput genetics to directly demonstrate that additional gene copies or the gain of a new gene cluster is sufficient to confer protection against bile salts.

### Gene hits do not consistently confer a benefit in *E. coli*

A key feature of our platform is developing an overexpression system in a gut anaerobe that is physiologically similar to the Bacteroidales assayed in our fitness screen. To test if *B. theta* is a more suitable expression host for detecting beneficial genes than the commonly used *E. coli*, we tested six of our validated gene hits in *E. coli* MG1655. This consists of ɑ-glucosidase in palatinose, hexokinase in d-glucosamine, *Pj*-RafT in raffinose and FOS, 2-ketoisovalerate oxidoreductase in ceftriaxone, FabX in ceftriaxone, and the alternative lipid biosynthetic cluster in cholate (Supplementary Fig. 9). Overexpression of hexokinase in *E. coli* delayed growth in d-glucosamine, but supported a higher final optical density. RafT was slightly beneficial for growth on FOS, but not raffinose, which may reflect differences in enzyme activities for the hydrolysis of each oligosaccharide critical for metabolism. 2-ketoisovalerate oxidoreductase conferred an increased tolerance toward ceftriaxone. However, no growth benefit was observed in the remaining hits. Overall, among the six genes overexpressed in *E. coli*, a fitness gain was observed in three of seven assayed conditions (Supplementary Fig. 9). Our data support the use of *B. theta* as an expression platform for phylogenetically and physiologically similar gut anaerobes.

## Discussion

Boba-seq is a new platform for constructing barcoded overexpression libraries for scalable and quantitative phenotypic screens. Here, we generated seven libraries using genomic DNA from *Bacteroides* and *Parabacteroides* species, an important clade of human gut bacteria. First, we validated our method in a complementation screen. Next, using wild-type *B. theta* as the expression host, we identified significant phenotypes in carbon utilization and stress tolerance across 120 unique regions and 22 compounds (Supplementary Data 5). By assaying DNA from multiple bacteria, we identified 30 hits supported by multiple protein homologs, which provided further confidence in our results.

Overall, we improved annotations and/or obtained new phenotypes for 29 protein clusters. We identified an outer membrane transporter for the uptake of raffinose, a plant-derived carbohydrate that is non-digestible by humans. This marks the first report of raffinose transporters in Bacteroidales. Using biochemistry and genetics, we uncovered a new d-glucosamine hexokinase. A region encoding an ssDNA-binding protein within a MGE led to genomic recombination and a growth advantage on l-fucose, a key nutrient in the gut, although the precise mechanism remains unclear. Furthermore, we discovered genes that enhance tolerance toward antibiotics and bile salts, prevalent stressors encountered in the gut. Among these, genes involved in lipid biosynthesis, including a *Parabacteroides*-associated gene cluster absent in *B. theta*, increased tolerance to a cephalosporin or to bile salts. An epimerase that likely alters surface polysaccharides was also beneficial in bile salts.

The selection of a physiologically related genetic background is critical for phenotypic assays. In support of this, we tested six validated gene hits in *E. coli* across seven conditions and only observed growth advantages in three conditions (Supplementary Fig. 9). In additional to major metabolic differences between these two bacteria, the cell surface of Bacteroidales is distinct from *E. coli* and consists of numerous lipoproteins dedicated to glycan degradation. Moreover, Bacteroidales display a wide range of surface architectures with diverse combinations of capsular polysaccharides where composition is controlled through phase-variation^28^.

We demonstrate the benefit of combining overexpression data with loss-of-function data to strengthen gene-to-phenotype mappings. 10 protein clusters have RB-TnSeq fitness data that are consistent with our Boba-seq data (Supplementary Data 5)^10,11^. The scalability of both loss-of-function and gain-of-function libraries using barcoding techniques for Bacteroidales is an exciting development. By constructing RB-TnSeq and Boba-seq libraries for a large number of isolates and screening across identical conditions, we can rapidly generate specific functional hypotheses for uncharacterized genes.

A few variables can be adjusted in our workflow. Current libraries have fragment lengths of ~3 kb and may not capture pathways with several proteins. Therefore, insert size can be increased to cover larger gene clusters. Additionally, the nature of competitive growth assays can lead to “jackpot” effects where the most fit strains outcompete other strains with a beneficial fragment and reduce the number of hits. This effect can be reduced by increasing the number of replicates, as BarSeq only costs a few dollars per sample, or by assaying libraries on agar plates or in microfluidic droplets to spatially separate strains and prevent competition^75,76^. Spatial separation of cells could also allow for the detection of secreted proteins that would otherwise increase the fitness of surrounding cells in a pooled assay format.

Boba-seq can be readily scaled. Our empty vector library has ~14 million barcodes and although we multiplexed six libraries with ~300,000 total strains, this can be increased. Libraries can be built using DNA from complex samples to access greater genetic diversity and uncultivated taxa. Moreover, our vectors replicate in at least nine additional Bacteroidales besides *B. theta*, which may provide more suitable host genetic backgrounds to screen for specific phenotypes. In addition to using wild-type strains as expression hosts, deletion mutant strains can be used for high-throughput complementation screens. Finally, Boba-seq libraries can be tested in additional assay formats, including host colonization, phage infection assays, suppression of toxic genes, and adhesion^12,25,26,50,77,78^.

## Methods

### Strains and cultivation conditions

Strains, plasmids, and primers used in this study are listed in Supplementary Data 1. *E. coli* strains were routinely cultured in Luria-Bertani Lennox (LB) with the appropriate selection using 100 μg/mL ampicillin (Amp100), 50 μg/mL carbenicillin (Carb50) or 50 μg/mL kanamycin (Kan50) at 37 °C with shaking at 200 rpm. *E. coli* WM3064 was grown with 300 μM diaminopimelic acid (DAP) supplement. Species within the order Bacteroidales were grown at 37 °C in an anaerobic chamber (Coy) maintained with a gas mix of 10% H_2_, 5% CO_2_, 85% N_2_. Media recipes and commercial sources of substrates are provided in Supplementary Data 4. Routine culturing was carried out in supplemented Brain Heart Infusion (BHIS) where BD Bacto Brain Heart Infusion was freshly supplemented with 5 mg/L hemin and 2.5 μL/L vitamin K_1_. The Varel-Bryant (VB) defined minimal medium was used for the majority of complementation and fitness assays as well as validation growth curves^38^. B12 vitamin was excluded and instead, methionine was used in our medium. Cysteine was substituted with dithiothreitol (DTT) as the reducing agent for certain carbon conditions. An exception to this was the heparin validation growth curves, which were obtained in another defined minimal medium (MM)^79^. Media were made anaerobic by pre-reducing in the anaerobic chamber at least one day prior to use. Conjugation mixes were selected on 200 μg/mL gentamicin (Gm200) and 5 μg/mL erythromycin (Erm5) to obtain the correct mutant.

### Construction of replicative vectors for *B. theta*

The pNBU2_erm-TetR-P1T_DP-GH023 integrative vector was modified to generate replicative vectors. This vector contains a previously reported anhydrotetracycline (aTc) inducible promoter and RBS (P1T_DP-GH023)^33^. A PmlI restriction site, the synthetic terminator L3S2P21, and BsmBI sites used for barcoding were inserted downstream of the inducible promoter-RBS cassette^29,34,35^. First, a BsmBI restriction site was removed from the pNBU2_erm-TetR-P1T_DP-GH023 vector using site-directed mutagenesis. The pNBU2 vector with no BsmBI site was linearized by PCR to exclude the attachment sites. Fragments containing replicative machinery and origin of replication from pTIO-1, pKSH24, and pFD340 were PCR amplified and purified using GFX DNA and Gel Band Purification kit^30–32^. The third gene block consisted of an insertion site, a terminator, and a barcoding site^10,34,35^. Three-part Gibson Assembly reactions were performed to generate pNBU2_repA1, pNBU2_repA2, and pNBU2_repA3 containing portions from pTIO-1, pKSH24, and pFD340, respectively. Gibson reactions were carried out according to manufacturer’s recommendations (NEB, Gibson Assembly master mix). This was transformed into electrocompetent *E. coli* EC100D *pir+* and plated onto LB-Carb50 to obtain the correct construct. All vectors were confirmed by Sanger sequencing.

### Detection of inducible expression using Luciferase assays

To quantify gene expression on the three pNBU2_repA vectors, luciferase assays were performed as previously reported, using Promega’s Nano-Glo Luciferase Assay System Kit^33^. Briefly, the NanoLuc gene was inserted downstream of the aTc-inducible promoter via Gibson assembly. Sequence-verified vectors were conjugated into *B. theta*. These *B. theta* strains were inoculated into BHIS-Erm5 and grown anaerobically at 37 °C for 16–18 h. Starter cultures were subcultured into BHIS-Erm5 and varying concentrations of aTc at a 1:100 dilution to mid-exponential phase (OD of 0.4–0.6, 3–5 h). 500 µL of each culture were then pelleted by centrifugation at 5000 × *g*, and cells were lysed with 50 µL of 1× BugBuster Protein Extraction Reagent (Sigma). Lysates clarified at 21,130 × *g* for 5 min, and 10 µL of the supernatant was mixed with an equal volume of the NanoLuc reaction buffer containing the NanoLuc substrate. Luminescence was immediately measured using a BioTek Synergy H1 microplate reader (Agilent), and normalized to OD600 (optical density at 600 nm) of the culture at time of lysis.

### Determination of plasmid copy number using droplet digital PCR

Droplet digital PCR (ddPCR) was performed following a previously reported procedure to estimate the plasmid copy number of the pNBU2_repA vectors in *B. theta*^80^. Briefly, *B. theta* containing each vector was grown in BHIS-Erm5 for 16–18 h at 37 °C, followed by a 1:100 dilution and subculture to mid-exponential phase (OD of 0.7–0.9, 3–5 h). 500 µL of each culture were then harvested for DNA extraction using Qiagen’s DNeasy blood & tissue kit, after which 1 µg of DNA was digested using NdeI (NEB). ddPCR was performed using approximately 0.01, 0.1, or 1 ng of the digested DNA as template for each 40 µL reaction. PCR was performed using the Evagreen supermix and primers p-AH204-p-AH207 (Supplementary Data 1), following BioRad’s protocols for multiplexing with Evagreen and using an annealing temperature of 60 °C. Samples were analyzed using Bio-Rad’s QX200 droplet digital PCR system. The resulting data points were manually gated, and reported concentrations for each amplicon were used in determining the plasmid copy number.

### Barcoding of the pNBU2_repA1 vector

Barcodes were amplified from synthesized DNA using Phusion polymerase (NEB) at an annealing temperature of 58 °C, elongation time of 60 s, and 6 cycles^9^. pNBU2_repA1 vector was barcoded with random 20 nucleotides using Golden Gate Assembly. Each 20 μL reaction consisted of 1 μg of plasmid, 35 ng of barcode amplicons, 2 μL of T4 DNA ligase buffer, 1 μL of T4 DNA ligase (NEB), and 1 μL of BsmBI/Esp3I enzyme (Thermo). This resulted in a 2:1 molar ratio of insert to vector. Six reactions were incubated at 37 °C for 5 min and 16 °C for 10 min, which were repeated 10 cycles, followed by 37 °C for 30 min. All reactions were pooled, cleaned across two columns from the DNA clean and concentrator kit (Zymo), and eluted twice with 10 μL H_2_O. Uncut vectors were removed with additional incubations with BsmBI/Esp3I in 25 µL reactions according to the manufacturer’s protocol. Reactions were incubated at 37 °C for 2 h, after which 1 µL of BsmBI/Esp3I was added for incubation at 37 °C for 16 h. Each reaction was cleaned using DNA clean and concentrator kit (Zymo) and eluted twice with 8 μL H_2_O. 7 μL of cleaned reaction was transformed into 50 μL of electrocompetent *E. coli* WM3064 for a total of 4 transformations. All aliquots were recovered in 7 mL S.O.C. medium with 300 µM DAP for 1.5 h. An aliquot was removed for CFU counting and confirmed high transformation efficiency of 10^6^–10^7^ CFUs per mL. Recovered cells were inoculated into 1 L of LB (50 μg/mL Carb, 300 μM DAP) for selection. Each vector library was purified for BarSeq to determine barcode diversity.

### Construction and quantification of barcoded genomic libraries

gDNA was purified from Bacteroidales using a DNeasy UltraClean Microbial Kit or DNeasy Blood & Tissue Kit (Qiagen). Up to 2 μg in 200 μL TE buffer was sheared by ultrasonication to an average of 3–5 kb with a Covaris S220 focused ultrasonicator using red miniTUBEs. Sheared gDNA was then gel-purified and size-selected by cutting out the 3–6 kb range. 4 µg of barcoded pNBU2_repA1 was digested in 50 µL reactions with 4.5 µL of PmlI. This was incubated at 37 °C for 30 min. Linearized vectors were dephosphorylated with 1 µL of rSAP (NEB) at 37 °C for 10 min and then deactivated at 65 °C for 5 min. Digested vectors were gel-purified using the Monarch DNA Gel extraction kit.

The *B. theta* library was constructed using blunt-end ligation, however vector rearrangement issues were observed by Sanger sequencing of individual *E. coli* clones. Cloning steps taken are similar to the previously reported Dub-seq library^24^. Briefly, sheared and size-selected gDNA from *B. theta* was end-repaired using End-IT DNA End-Repair kit (Lucigen) in a 50 µL reaction consisting of 2 µL End-It Enz mix, 5 µL dNTP mix, 5 µL 10 mM ATP, 5 µL buffer, and 4 µg gDNA. This was incubated at room temperature for 45 min and deactivated at 70 °C for 20 min. This was gel-purified and size-selected for 3–6 kb fragments. The end-repaired, sheared DNA fragments were then ligated to linearized and dephosphorylated barcoded vectors. A 20 µL ligation reaction was performed with 10 µL Blunt/TA ligase master mix (NEB), 100 ng of vector backbone, and 4-fold molar excess of end-repaired DNA fragments, and incubated at room temperature for 20 min. Reaction was purified using Monarch PCR & DNA cleanup kit and eluted twice with 10 µL H_2_O. 20 ng of cleaned ligation reaction was transformed into 50 µL aliquots of commercial *E.coli* EC100D that was diluted 4-fold with 10% glycerol. A total of 8 transformations were performed and each recovered in 1 mL of S.O.C. medium at 37 °C for 1.5 h with shaking at 200 rpm. An aliquot was removed for CFU counting to estimate library diversity. Recovered cultures were combined and inoculated into 500 mL of LB-Carb50 for selection. The final library was purified for BarSeq to obtain an estimated diversity. The initial *B. theta* library consists of ~440,000 barcodes and was diluted to obtain the final library with ~70,000 barcodes.

An alternative strategy was applied to the remaining six libraries, which did not lead to any observable vector rearrangement. They were constructed by cloning DNA fragments into the pCR™Blunt II-TOPO™ vector using Zero Blunt™ TOPO™ PCR Cloning Kit (Invitrogen), followed by PCR amplification of the inserts and Gibson assembly into barcoded pNBU2_repA1. In this protocol, sheared gDNA is first end-repaired in 20 µL reactions using Quick CIP (NEB) and following the manufacturer’s protocol. End-repaired inserts are column-purified. Ligations were carried out in 6 µL TOPO reactions that contained 0.5 or 1 µL of pCR II-Blunt-TOPO vector, 1 µL of salt solution, and 20 ng of end-repaired inserts. Reactions were incubated at room temperature for 30 min and cleaned using SPRI beads (Beckman Coulter). Briefly, 0.9 equivalent volume of bead solution was mixed with each TOPO reaction and incubated for 15 min. Beads were washed three times with 80% EtOH and allowed to dry briefly. DNA was eluted with 5 µL of H_2_O heated to 50 °C and transformed into 50 µL of electrocompetent 3-fold diluted *E. coli* EPI300 (Lucigen). An aliquot was taken out for CFU counting on LB-Kan50. Recovered cultures were stored as glycerol stocks at −80 °C. Multiple TOPO reactions and transformations were carried out until an estimate of ~40,000 to 100,000 CFUs was obtained for each library. Glycerol stocks of recovered cells from the same library were combined and inoculated into 200 mL of LB-Kan50 for selection. Inserts were PCR amplified from 150 ng of purified TOPO vectors using Q5 polymerase with an annealing temperature of 67 °C, 2 min extension, and 8 cycles. Template vectors were digested using DpnI and the 3–6 kb size range was gel-purified. Insert amplicons were ligated into barcoded pNBU2_repA1 in 20 µL of Gibson reactions using the manufacturer’s protocol. 20 ng of cleaned reaction was transformed into 50 µL of 4-fold diluted electrocompetent *E. coli* EC100D. Cells were recovered and stored as glycerol stocks before selection. Aliquots of the recovered cells were plated onto LB-Carb50 to estimate CFUs. Libraries were serially diluted to <100,000 CFUs and selected in liquid LB-Carb50 to make final glycerol stocks and perform BarSeq. All libraries were transformed into *E. coli* WM3064 for conjugation into *B. theta*.

Differences between libraries constructed using each cloning workflow were observed. The ligated *B. theta* library has the highest percentage of genes covered (98%) and the lowest strand bias. The PCR required for Gibson Assembly, though with a low cycling number, introduced biases that likely led to lower genome coverage values (Supplementary Data 2). Gibson-assembled libraries exhibited a slight strand bias toward fragments oriented opposite to the promoter (ratio of genes oriented opposite over genes oriented the same direction as vector promoter: 1.43–1.88). In contrast, the number of genes on either strand is very similar in the *B. theta* library, with a ratio of 0.98. This is likely a result of gene toxicity in *E. coli* caused by the lac promoter on the TOPO vector. This strand bias did not increase upon library conjugation into *B. theta*, which is consistent with the low basal expression of the aTc inducible promoter.

### PacBio sequencing to map libraries

Barcoded libraries purified from EC100D were used as templates for amplicon sequencing. Each sample was dual indexed using 5′-phosphorylated PCR primers containing 16 nt barcodes^81^. Barcode and insert regions were amplified in 50 µL PCRs with Q5 polymerase, 150 ng of vector template, 57 °C annealing temperature, 2 min extension time, and 6 or 8 cycles. 8–10 reactions per library were pooled, digested with DpnI twice (NEB), and column-concentrated. Amplicons were size-selected for >800 bp lengths using 0.55 equivalent volume of AMPure PB beads (PacBio). 200–600 ng of this was used as sample input for the manufacturer’s protocol: Preparing SMRTbell libraries using PacBio Barcoded Overhang Adapters for Multiplexing Amplicons. The universal adapter was used during the ligation step instead of a barcoded adapter since amplicons are dual indexed. Adapter-ligated amplicons were digested with exonuclease using SMRTbell Enzyme Clean Up Kit 2.0 and purified using 0.55 equivalent volume of AMPure PB beads. Final amplicon solutions were quantified using Qubit BR Assay and sizes were confirmed on the Bioanalyzer HS DNA chip (Agilent). Samples were sequenced on the PacBio Sequel II instrument using SMRT cell 8M (QB3 Genomics, UC Berkeley). See Supplementary Data 2 for the number of demultiplexed CCS reads used to map each library. In general, a good run on one PacBio SMRT cell 8M was sufficient to map 3–4 libraries.

### Computational pipeline used to map barcodes to genes

The Boba-seq script used to map barcodes to genes and detailed explanations can be found at https://github.com/OGalOz/Boba-seq and as a part of the deposited data. A summary is provided as Supplementary Fig. 2. Input data files are required and consist of: a reference assembly file (.fasta), a genome annotation file (.gff/.gff3), and long reads data (.fastq). The user also needs to provide a fasta file containing 4 short oligo sequences that flank the barcode and the insert fragment on each amplicon. A configuration.JSON file is used to set parameters for each step. Briefly, reads were first demultiplexed using PacBio’s lima tool. Barcode and insert sequences are extracted using 15 bp flanking oligos using usearch (www.drive5.com/usearch/). Filters are applied to exclude reads with concatemers and incorrect barcode lengths. Inserts with an expected error of >10 are excluded using vsearch (https://github.com/torognes/vsearch). Inserts are mapped to the reference genome using minimap2 and results are kept for hits with high percent coverage and percent match values^82^. The majority of barcodes mapped to a single genomic location. For barcodes that are mapped to multiple locations, most are due to sequencing errors and are within a few nt of each other. For barcodes mapping to overlapping locations, the location with the top read count is kept. Barcodes mapped to distant genomic locations are kept in the mapping tables but are excluded from our fitness analysis. Finally, genomic positions are mapped to protein-coding genes to generate mapping tables, which are used to calculate library statistics.

### Conjugation into *B. theta* and other Bacteroidales

Single vectors were first transformed into conjugation donor strains *E. coli* S17-1 or WM3064. 40 mL of Bacteroidales recipient at OD ~ 0.1 was mixed with 4 mL of *E. coli* donor at OD ~ 1 outside of the anaerobic chamber. Mixed cultures were pelleted by centrifugation and resuspended in 250 μL BHIS. Each cell mixture was then pooled onto BHIS or BHIS-DAP (for WM3064) plates and incubated at 37 °C overnight aerobically for >16 h. The entire cell mass was then scraped and resuspended in 1 mL of BHIS, from which 20–100 µL was plated onto BHIS-Gm200-Erm5 agar. Antibiotic plates were incubated at 37 °C anaerobically for 48 h or longer until distinct colonies formed. Single colonies were restreaked onto BHIS-Gm200-Erm5 once, from which a single colony was then inoculated into BHIS-Gm200-Erm5. Bacteroidales cultures were inoculated into LB and incubated aerobically at 37 °C to ensure a lack of *E. coli*. Strains were checked by Sanger sequencing of plasmids and 16S rRNA. pKSH24 and pTIO-1 were conjugated into *B. theta* to assess replication in a new host. pNBU2_repA1, pNBU2_repA2, and pNBU2_repA3 with NanoLuc were conjugated into 13 different *Bacteroides*, *Parabacteroides*, and *Phocaeicola* species.

### Complementation Boba-seq libraries and assays

*B. theta, B. uniformis, B. fragilis*, and *P. johnsonii* Boba-seq libraries were conjugated into *B. theta* Δ*tdk*ΔBT2158 or Δ*tdk*ΔBT3703 using *E. coli* WM3064 as the conjugation strain. 200 µL of conjugation mix in 1 mL BHIS was inoculated into 100 mL of BHIS-Gm200-Erm5 anaerobically. Each conjugation mix was serially diluted for CFU counting to ensure that ~10^6^ transconjugants were inoculated for selection. Cultures at OD > 1 were pelleted and resuspended in BHIS with 0.15% Cys and 15% glycerol to make glycerol stocks.

For complementation assays, library starter cultures were diluted to an OD of 0.5 in BHIS-Gm200-Erm5 with 40 ng/mL aTc and incubated at 37 °C for 3.5 h to induce gene expression. Cells were pelleted and washed with VB minimal medium twice; these served as Time0 samples. Each library was inoculated at a starting OD of 0.02 in 1 mL VB-Erm5-aTc40 and 20 mM of either leucrose, palatinose, trehalose, or ɑ-cyclodextrin. Cultures were incubated at 37 °C and harvested when turbid. Each assay was also streaked onto BHIS-Gm200-Erm5 agar plates to isolate individual strains. Colony PCR and Sanger sequencing were used to identify clones containing BT2158, BT4448, or BACUNI_RS05440.

### Growth assays of complemented mutants

*B. theta* mutants complemented with BT2158, BT4448, or BACUNI_RS05440 (Supplementary Data 7), Δ*tdk*ΔBT2158, and Δ*tdk* parental strain were inoculated into BHIS-Erm5 or BHIS and grew overnight at 37 °C. Cells were pelleted and washed once with VB minimal medium. Each mutant was inoculated at a starting OD of 0.05 in 200 µL VB-Erm5 and 20 mM trehalose. *B. theta* Δ*tdk* and Δ*tdk*ΔBT2158 were inoculated in the same manner, except without Erm. Growth measurements were obtained in a 96-well plate using an Epoch 2 Microplate Spectrophotometer (Agilent) inside an anaerobic chamber. OD600 values shown on plots are pathlength-corrected and blank-normalized.

### Fitness screen of pooled libraries in wild-type *B. theta*

*B. caccae, B. fragilis*, *B. salyersiae, B. uniformis, P. johnsonii*, and *P. merdae* libraries were conjugated into wild-type *B. theta* as described for complementation libraries. For fitness assays, 1 mL of glycerol stock was pelleted and resuspended in 10 mL of BHIS-Erm5 with 40 ng/mL of aTc as starter cultures to grow for 16–18 h. These were then pelleted and washed with VB-Erm5 twice. Libraries were pooled equally based on OD to a final OD of 5, which served as Time0 samples. For carbon utilization assays, pooled libraries were inoculated at a starting OD of 0.04 in 1 mL of VB-Erm5 with or without 40 ng/mL aTc, and a single carbon substrate at the concentration listed in Supplementary Data 4. For stress tolerance assays, pooled libraries were inoculated at a starting OD of 0.1 or 0.04 in 0.5 mL of BHIS-Erm5, 40 ng/mL aTc, and an inhibitory compound at a range of concentrations listed on Supplementary Data 4. Assays were carried out in 48-well plates and incubated at 37 °C. Each condition was set up in replicates of two on the same day. A few conditions were repeated on a second day of experimentation where new conditions were tested as well. Cultures were harvested when turbid for BarSeq.

### DNA barcode sequencing (BarSeq) and analysis

Boba-seq libraries were purified from *E. coli* using E.Z.N.A. Plasmid DNA Mini Kit (Omega Bio-tek) or Monarch Plasmid Miniprep kit (NEB). However, this led to low yields and high levels of contaminants when extracting from Bacteroidales. DNA purified from the same set of Bacteroidales samples using plasmid purification kits and genomic DNA purification kits were tested for barcode PCR amplification. BarSeq results from both purified plasmids and gDNA were comparable and therefore, DNeasy Blood & Tissue Kit or QiaAmp DNA QIAcube HT Kit (Qiagen) for 96-well sample plates were used to extract DNA from *B. theta* cultures for BarSeq for all fitness assays.

Barcodes were amplified using the previous-published “BarSeq_V3” primers or using new “BarSeq_V4” primers (Supplementary Data 1)^9,83^. The BarSeq version 4 primers are similar to the BarSeq version 3 primers but use a 10-bp index sequence for P7 primers and a 8-bp index for P5 (instead of 6 bp for both in version 3) and allow multiplexing up to 768 samples (instead of 96). Briefly, BarSeq PCRs were performed as 50 µL reactions with Q5 polymerase (NEB) and 200 ng of template DNA: 98 °C for 4 min, 25 cycles of 98 °C for 30 s, 55 °C for 30 s, and 72 °C for 30 s, and 72 °C for 5 min. For both BarSeq3 and BarSeq4 designs, the P7 (or P2) BarSeq primers allowed demultiplexing by Illumina software^9^, and the P5 (or P1) BarSeq primers contained an additional index checked by the MultiCodes.pl script^84^. All PCRs were pooled equally in volume and column-cleaned. Up to 96 samples (BarSeq3) or 152 samples (BarSeq4) were multiplexed for sequencing on full lanes of Illumina HiSeq4000 (QB3 Genomics, UC Berkeley; Novogene), partial lanes of NovaSeq6000 (Novogene), or full lanes of MiSeq (v2 reagent kit). 11,000–94,000 reads per sample were obtained for complementation assays sequenced on the MiSeq and 0.3–5.3 million reads were obtained for samples sequenced on the HiSeq or NovaSeq.

BarSeq reads were converted to counts per barcode using the MultiCodes.pl script in the feba code base (https://bitbucket.org/berkeleylab/feba/), with the -minQuality 0 option and either -bs3 or -bs4 for each version of BarSeq primers. To estimate the diversity of barcoded libraries, only reads that had a quality score of ≥30 at each position (the -minQuality 30 option) were used, which corresponds to an error rate for barcodes of at most 0.001 * 20 nt = 2%. Furthermore, any barcodes that were off-by-1 errors from a more abundant barcode were eliminated. Barcodes observed in the PacBio dataset are compared to barcode sequences from the BarSeq data to determine the percentage of barcodes mapped.

For the highly diverse empty library, the Chao1 estimator was used to estimate the barcode diversity from 2.99 million high-quality reads^83^, The Chao1 estimator uses the number of barcodes seen just once, but some may be due to sequencing errors. To correct for this, barcodes that were off-by-1 nucleotide from a more abundant barcode were ignored, and the number of singletons was reduced by an assumed error rate ranging from 0% to 2%. This yielded estimates ranging from 13.5 to 14.1 million barcodes for the empty pNBU2_repA1 library.

### Calculation of fitness scores for each fragment

Only barcodes that mapped to a single location in one library and that were present in the conjugation donor (*E. coli* WM3064) libraries were considered. 2% of barcodes mapped to more than one library and another 5% of mapped barcodes were not detected in the BarSeq data for the conjugation donor libraries. For complementation assays, we computed fitness scores for all of these fragments. For fitness assays in the wild-type *B. theta* background with six pooled libraries, 280,036 of the 305,382 barcodes (that were present in WM3064) were detected in the Time0 samples. Also, barcodes that were not detected in Time0 samples occasionally had statistically significant benefits. This is likely due to positive selection that led mutants to gain in abundance in the assay condition despite their low initial abundance. Therefore, fitness scores were computed for barcodes that were confirmed to be present in the *B. theta* wild-type background with at least 10 reads total across all experimental and Time0 samples. The script used to calculate fitness scores can be found at https://github.com/morgannprice/BobaseqFitness.

The fitness of each strain was defined as:

$${\log }_{2}\{(n+{{{\rm{\psi }}}})/N\}\,-\,{\log }_{2}\{\,({n}_{0}+1/{{{\rm{\psi }}}})/{N}_{0}\}$$

$${{{\rm{\psi }}}}={{{\rm{sqrt}}}}({N/N}_{0})$$

where n is the number of reads for this barcode in the experimental sample, n_0_ is the number of reads for this barcode across the Time0 samples, and N and N_0_ are the total number of reads for all considered barcodes in the experimental or Time0 samples, respectively. The pseudocount ψ is added to the counts to ensure that the log ratio is not undefined if either count is zero. This value of ψ also ensures that if both counts (n and n_0_) are 0, then the fitness will be zero (corresponding to no evidence of a change in relative abundance). If the experimental and Time0 samples have the same total number of reads, then ψ = 1.

### Determination of statistically significant hits

The minimum fitness value threshold for statistical significance was selected based on a control comparison between the two Time0 samples collected on the same day for the large fitness screen. Each sample contained a mixture of six genomic libraries and had 2.9 million or 3.0 million total reads. Of the 305,382 barcodes considered, the fitness values ranged from −3.9 to +4.1. Therefore, a minimum fitness value of +5 was selected for a statistically significant hit or barcode.

Because a few complementation assays were sequenced at much lower coverage, a z score cutoff was also applied. The standard error of the fitness value was estimated as the square root of the variance given Poisson noise in counts^9^, or

$${{{\rm{\sigma }}}}={{{\rm{sqrt}}}}\{1/(n+{{{\rm{\psi }}}})+1/({n}_{0}+1/{{{\rm{\psi }}}})\,\}/{{\mathrm{ln}}}(2)$$

The z score is then fitness/σ and z ≥ 4 is selected as the cutoff. For the standard normal distribution and 305,382 barcodes, 10 values would be expected above this threshold.

### Selection of biologically consistent hits and the likely causative protein for each region

We focused on barcodes and inserts that had significantly high fitness scores in both replicate experiments conducted in the same condition and on the same day. We considered these barcodes to have a biologically consistent phenotype if either an overlapping fragment or a fragment encoding a similar protein had statistically significant benefits. Specifically, an insert was confirmed by overlap if another insert from the same region of the genome was significant in both replicates. An insert was confirmed by similarity if the likely causative protein for the region (see below) was similar (at least 40% identity and 75% coverage) to a protein that was found within another insert that had significantly high fitness in two replicates for the same condition. For experiments in the wild-type *B. theta* background where libraries were pooled, the confirmation would occur in the same assays. For complementation assays where libraries were assayed separately, the criterion might be fulfilled from different assays in the same condition.

For each region with inserts that were statistically significant in both replicates, the insert with the highest fitness score (after averaging replicates) was selected first. For each protein encoded within this insert, we computed several scores. First, we counted the number of significantly beneficial inserts that contain this protein (with each replicate counted separately). Second, we counted the number of similar proteins that were in high-scoring regions. Third, we computed the average fitness score for all inserts containing this gene. To select the likely causative protein for each region, we chose the one with the highest number of beneficial inserts, or the highest number of similar proteins, or the highest average fitness. We used the additional scores to break ties.

For each of the putative causative proteins, we visually inspected the plot of fitness scores versus insert fragments. When available, we also considered the plots for similar experiments (such as the same carbon source with a different base medium, or a different concentration of the stress, or the same condition conducted on a different day) with similar protein hits. In 20 cases, we determined that more than one protein is necessary to confer a strong benefit. In 6 cases, we identified a different protein as beneficial than the initial selection based on scores. In 28 cases, we were not sure which protein(s) conferred the benefit and left these hits uncurated in Supplementary Data 5. For the remaining 162 cases, the automatically selected protein was deemed correct.

### RB-TnSeq library construction in *Phocaeicola vulgatus* CL09T03C04 and fitness screen

A barcoded transposon mutant library was constructed in *P. vulgatus* CL09T03C04 using a previously described barcoded vector (pTGG46_NN1) and methodology to what was previously applied to *B. theta* VPI-5482^11^. To ensure that the recipient was in late log phase at the time of conjugation, an overnight culture of *P. vulgatus* CL09C03T04 was subcultured 1:20 into 50 mL of BHIS_K3 (BHIS as described by ref. ^11^. supplemented with 1 mg/L vitamin K3). Transposon mutagenesis was carried out at a 1:1 OD ratio of *E. coli* WM3064 donor with pTGG46_NN1 (AMD776 library) at 0.9 OD (log phase) and *P. vulgatus* CL09T03C04 recipient (log phase). Conjugation was carried out on four 0.45 μm gravimetric analysis membrane filters (Millipore) on BHIS_K3 plates supplemented with DAP for 16 h. The conjugation mix was plated onto BHIS_K3 plates supplemented with 5 μg/mL erythromycin and 100 μg/mL gentamicin and incubated at 37 °C for two days. Around 150,000 transconjugants were scraped together and outgrown in BHIS_K3 supplemented with 5 μg/mL erythromycin and 100 μg/mL gentamicin to further counter select against the *E. coli* donor. Upon saturation, multiple tubes were frozen down in glycerol as the final mutant library (Bvulgatus_CL09T03C04_ML5). Genomic DNA was extracted using the DNeasy blood and tissue kit (Qiagen) and 157,372 uniquely barcoded mutants were confidently mapped by TnSeq^9,11^. Genome-wide fitness assays were performed as described for *B. theta* VPI-5482^11^, and gene fitness values were calculated as previously described^9^. BarSeqR.pl from the feba code base (https://bitbucket.org/berkeleylab/feba) was used to compute gene fitness values and t-like test statistics in Supplementary Data 6.

### Validation of gene hits in *B. theta*

Selected gene hits from the fitness screens were PCR amplified from the corresponding gDNA, cloned into the pNBU2_repA1 backbone via Gibson Assembly, and transformed into *E. coli* S17-1 to generate validation vectors. Mutants with high fitness scores were selected for validations and the exact fragment boundaries for these were extracted from library mapping tables to design primers (Supplementary Data 7). Additionally, amplicons of the precise gene or genes were obtained. Validation vectors were conjugated into *B. theta*, and each strain was grown in the same condition as carried out for the fitness assay with AraC hits being an exception. Here, we switched to a different defined minimal medium (MM) where *B. theta* grew more quickly in heparin (see Supplementary Data 4 for composition)^79^. Each strain was cultured in 200 µL on a 96-well plate in replicates of 3. In a few cases, the empty vector control was grown up in replicates of 2. Growth measurements were obtained using an Epoch 2 Microplate Spectrophotometer (Agilent). OD values are pathlength-corrected and media blank-normalized.

### Validation of gene hits in *E. coli* MG1655

Selected gene hits from the fitness screens were cloned into BamHI-linearized pTrcHis via Gibson Assembly for expression without any tag. *E. coli* TOP10 or 10-beta strains were used for cloning. The pTrcHis vector is a modified version that lacks the C-terminus His tag. Sequence-verified plasmids were then transformed into *E. coli* MG1655 for growth assays. Stress conditions were assayed in LB and carbon conditions were assayed in Difco M9 minimal medium, with 100 µg/mL ampicillin and 100 µM IPTG present for all conditions. Strains containing a gene hit or an empty vector were cultured in 200 µL on a 96-well plate in replicates of 3–4 for each condition. Multiple experiments were performed to support the observed phenotypes with data from one representative growth experiment shown in Supplementary Fig. 9. OD600 was measured using an Epoch 2 or a Synergy Microplate Spectrophotometer (Agilent). OD values are media blank-normalized.

### Overexpression and purification of hexokinase

Hexokinase (INE72_RS21610) was amplified from *B. caccae* CL03T12C61 and cloned into pET29b vector for protein overexpression and purification. 500 mL of *E. coli* BL21(DE3) transformed with pET29b-hexokinase was induced with 0.5 mM IPTG at OD ~ 0.6. Pellet was harvested and then resuspended in lysis buffer (50 mM Tris HCl pH 7, 100 mM NaCl, 1 mM PMSF, 0.2 mg/ml lysozyme, 1X SIGMAFAST protease inhibitor, 1% streptomycin sulfate, 5 mM BME). Resuspended cells were lysed using a sonicator probe. The clarified supernatant was loaded onto equilibrated Ni Sepharose 6 Fast Flow resin (Cytiva) and washed with a buffer containing 50 mM Tris HCl pH 7, 100 mM NaCl, and 5 mM BME. Protein was eluted using the wash buffer with 10, 20, 50, 100, or 200 mM imidazole and fractions were collected at each concentration. All fractions were visualized on SDS-PAGE and the appropriate fractions were pooled and concentrated down using 30 K MWCO concentrators. Concentrated protein eluate was desalted using a PD-10 column loaded with Sephadex G-25 resin (Cytiva). Protein concentrations were calculated using Abs280 measurements from Nanodrop and a molar extinction coefficient of 44,350 M^−1^ cm^−1^. In total, 4.2 mg of protein was purified to a concentration of 105 μM. Protein aliquots were stored at –80 °C.

### Hexokinase enzyme assays

In vitro coupled enzyme assays were performed to test substrate preference of hexokinase. Assay components include 50 mM Tris-Cl pH 7, 50 mM NaCl, 2 mM MgSO_4_, 1 mM NADH, 2 mM phospho(enol)pyruvic acid tri(cyclohexylammonium) salt (PEP), 1 mM ATP, and 1 mM substrate. Glucose, mannose, d-glucosamine, and N-acetyl-d-glucosamine were tested as substrates. Enzymes were added to concentrations of 10 U/mL lactate dehydrogenase from rabbit muscle (L2500-10KU, Sigma), 10 U/mL pyruvate kinase from rabbit muscle (P1506-1KU, Sigma), and 5 μM purified hexokinase. Lactate dehydrogenase and pyruvate kinase concentrations were also tested at a 2-fold increase to ensure they were in excess and not the rate limiting step. Assays were initiated by adding hexokinase and incubated for 5 min at room temperature. Assays were performed in volumes of 40 μL in triplicates in a 384-well plate. NADH consumption was observed through pathlength-corrected absorbance at 340 nm measured using a BioTek Synergy HTX plate reader (Agilent).

### RNA-seq analysis of *ssb* region in l-fucose

For RNA-seq experiments, a *B. theta* strain expressing the *ssb* fragment 2 (Supplementary Data 7) and a strain containing the empty vector were grown in BHIS-Erm5-aTc40 overnight. This was then inoculated into VB-Erm5-aTc40 with 20 mM l-fucose at an OD of 0.04. Each strain was cultured in replicates of 3 and grown to mid-exponential phase (OD600 = 0.4–0.8), which were harvested using RNAprotect bacterial agent (Qiagen). Total RNA was extracted using the RNeasy Mini kit (Qiagen), including on-column DNA depletion using the RNase-free DNase set (Qiagen). ~500 ng of total RNA from each sample was submitted to Novogene for further processing; this included rRNA depletion followed by library preparation with the NEBNext Ultra II Directional RNA Library Prep Kit for Illumina (NEB). Library quality was checked using a 2100 Bioanalyzer (Agilent).

7.5 to 10 M of PE150 reads were trimmed and aligned to the *B. theta* VPI-5482 genome (GCF_000011065.1) using HISAT2 (2.2.1) with the strand-specific option for paired-end reads, and default parameters otherwise^85^. Mapped reads were then assigned to genome features using featureCounts with the strand specific setting^86^. Due to concern over repeat regions, counts were generated with and without multimapping using featureCounts (part of Subread 2.0.1 package), which led to similar results. Final counts were generated with multimapping. These counts were normalized and analyzed using the DESeq2 package (1.38.2) in R (4.2.1), resulting in log2 fold-changes along with test statistics and adjusted *p*-values generated using the Wald test (Supplementary Data 8)^87^.

### Statistics and reproducibility

Statistical analyses for relevant experiments are described in the corresponding method sections in detail. No statistical method was used to predetermine sample sizes. The experiments were not randomized. The Investigators were not blinded to allocation during experiments and outcome assessment.

### Reporting summary

Further information on research design is available in the Nature Portfolio Reporting Summary linked to this article.

### Supplementary information

Supplementary Information

Peer Review File

Description of Additional Supplementary Files

Supplementary Data 1–8

Reporting Summary

## Data Availability

Plasmid maps, mapping tables for Boba-seq libraries, the input and output files from fitness analysis, and raw reads from the RNA-seq experiment are deposited on Figshare at: 10.6084/m9.figshare.24195054. RNA-seq data is also deposited on NCBI as BioProject PRJNA1045699. TnSeq and fitness data from the *P. vulgatus* CL09T03C04 RB-TnSeq library is deposited on Figshare at: 10.6084/m9.figshare.25620471.v1, which can also be accessed through the Fitness Browser (https://fit.genomics.lbl.gov/). Bacteroidales plasmids are available on addgene (ID 211910-211912). Please direct all data and material requests to Y.Y.H. and A.P.A.

## Code availability

The script for mapping Boba-seq libraries is available at https://github.com/OGalOz/Boba-seq. The script for calculating fitness scores is available at https://github.com/morgannprice/BobaseqFitness. A snapshot of the scripts used for library mapping and fitness score calculations are deposited at: 10.6084/m9.figshare.24195054.
