# Supplementary material for: Barcoded overexpression screens in gut Bacteroidales identify genes with roles in carbon utilization and stress resistance
Source: Nat Commun. 2024 Aug 5;15:6618. doi: 10.1038/s41467-024-50124-3 (PMC11300592; doi:10.1038/s41467-024-50124-3)
Supplement: Supplementary file 1 — Supplementary Information [file 41467_2024_50124_MOESM1_ESM.docx]

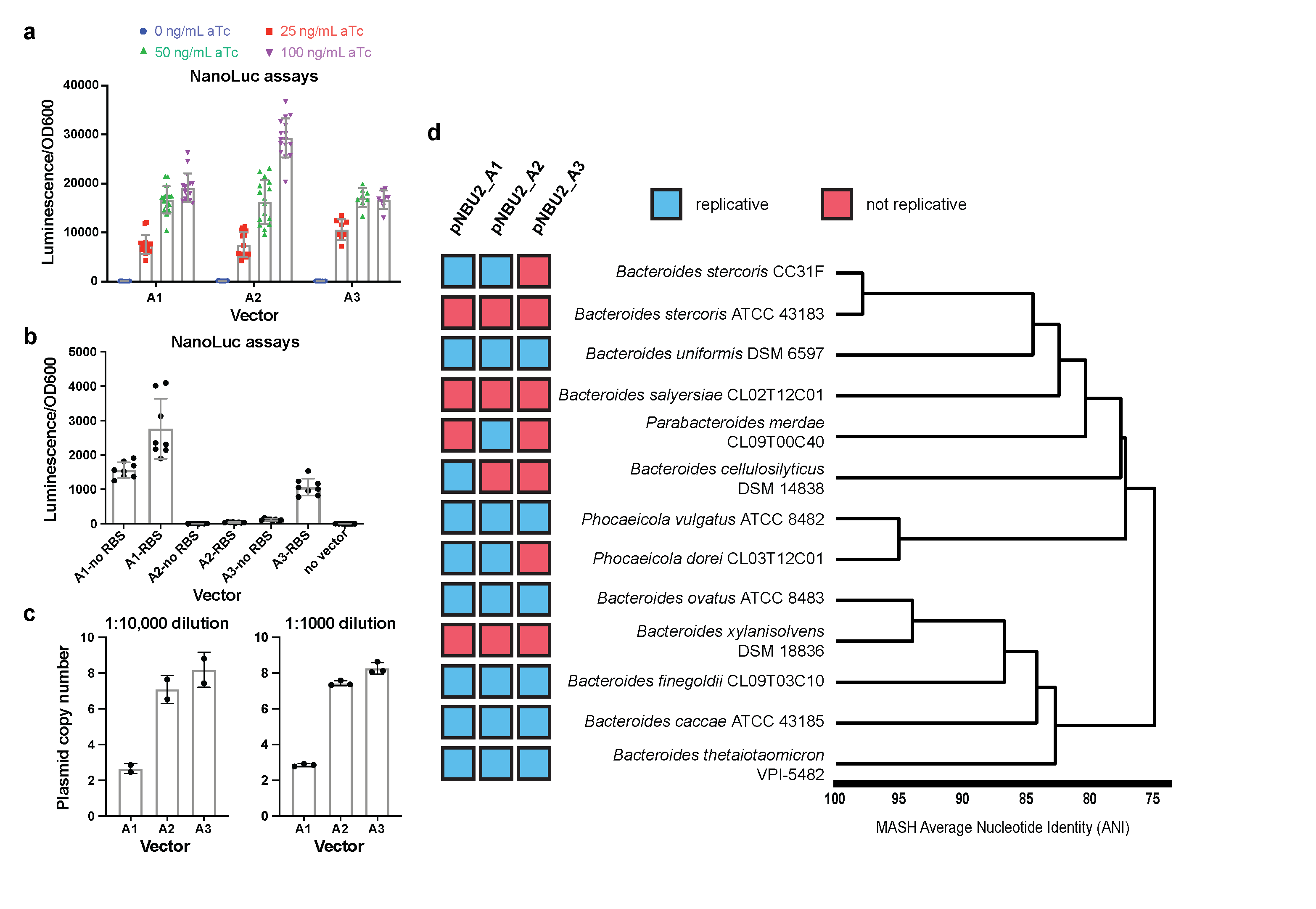


##### **Supplementary Figure 1.** Anhydrotetracycline (aTc) induces expression of NanoLuc on pNBU2 replicative vectors in *B. theta*. A) A range of aTc concentrations (0, 25, 50, 100 ng/mL) was tested for three vectors with NanoLuc inserted downstream of the inducible promoter and an RBS. A total of two or four experiments were performed per vector with each experiment consisting of four replicates. Data are shown as mean +/- standard deviation. B) NanoLuc was inserted into the pNBU2_repA1 vector in the opposite orientation to the aTc inducible promoter to test for constitutive promoter activity downstream of the insert site. NanoLuc gene with and without the upstream RBS (GH023) were tested in the absence of aTc. Two experiments were carried out with each experiment consisting of four replicates. Luminescence values were normalized by OD600 for all assays. Data are shown as mean +/- standard deviation. C) Plasmid copy numbers in *B. theta* determined using droplet digital PCR (ddPCR). Cultures in the late exponential phase were harvested for this assay. Replicates from two dilutions were used to calculate plasmid copy numbers. Data are shown as mean +/- standard deviation. D) Host range of pNBU2-based vectors across 13 Bacteroidales. Genome distance estimation and visualization were performed using dRep.^90^

#####
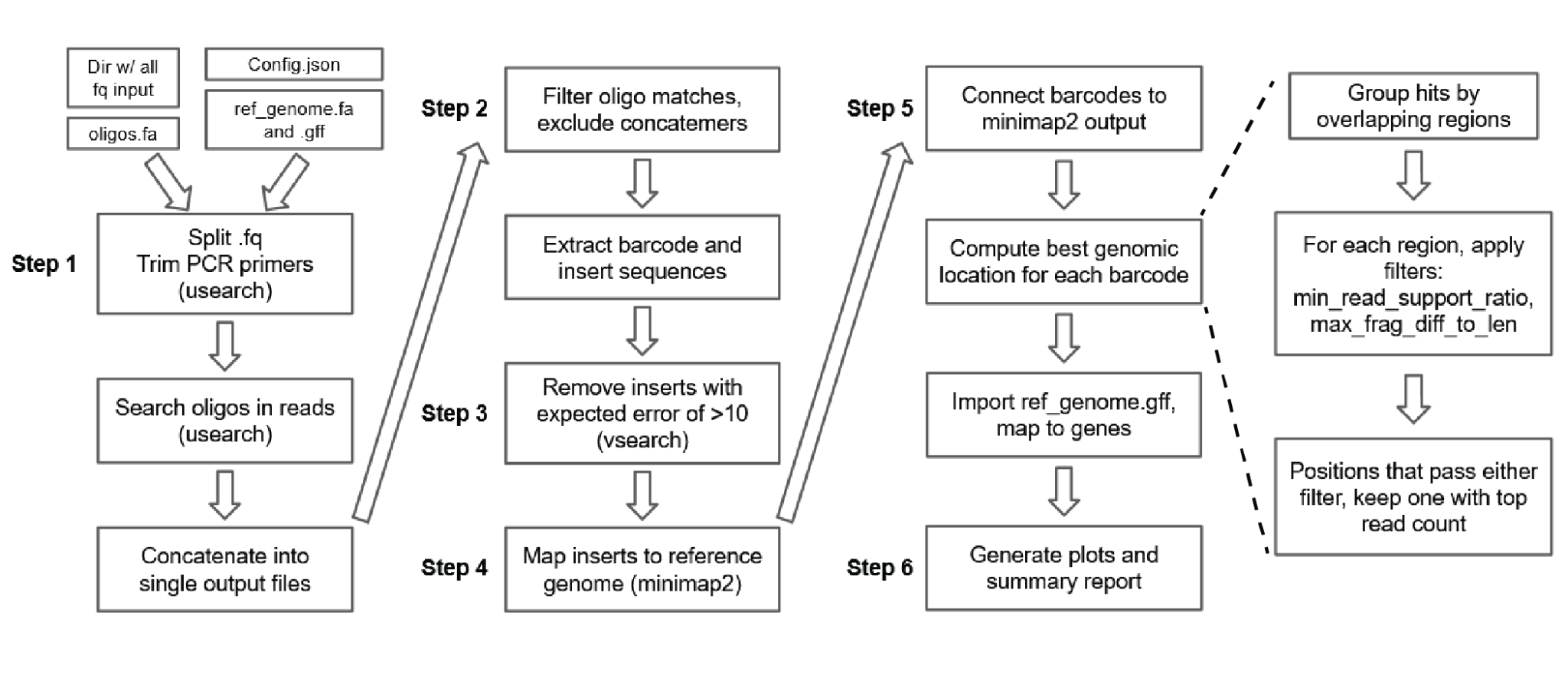
**Supplementary Figure 2.** Overview of the Boba-seq mapping script. Input files, tools, and all steps are listed. Additional details for step 5 are listed for barcodes that map to multiple, genomic regions. Although the empty vector library has a high barcode diversity, it is possible to clone multiple inserts into vectors sharing the same barcode. We observed this in a very small fraction of barcodes and later removed them from our fitness score analysis, but they are reported by the mapping script. Two parameters are used to compute the best genomic location for barcodes that map to very similar positions. First, the ratio of highest read count to the second highest read count (min_BC_support_ratio value) is set at a threshold of 3 to identify the position supported by more reads. Second, the max_frag_diff_to_len value is the fraction of the maximum distance between start and end positions of 5’ of mapped locations plus the maximum distance between start and end positions of 3’ of mapped locations within each region, over the average insert length. This is set to a threshold of 0.25 in our current pipeline. All filters and parameters can be readily adjusted in the configuration .JSON input file.


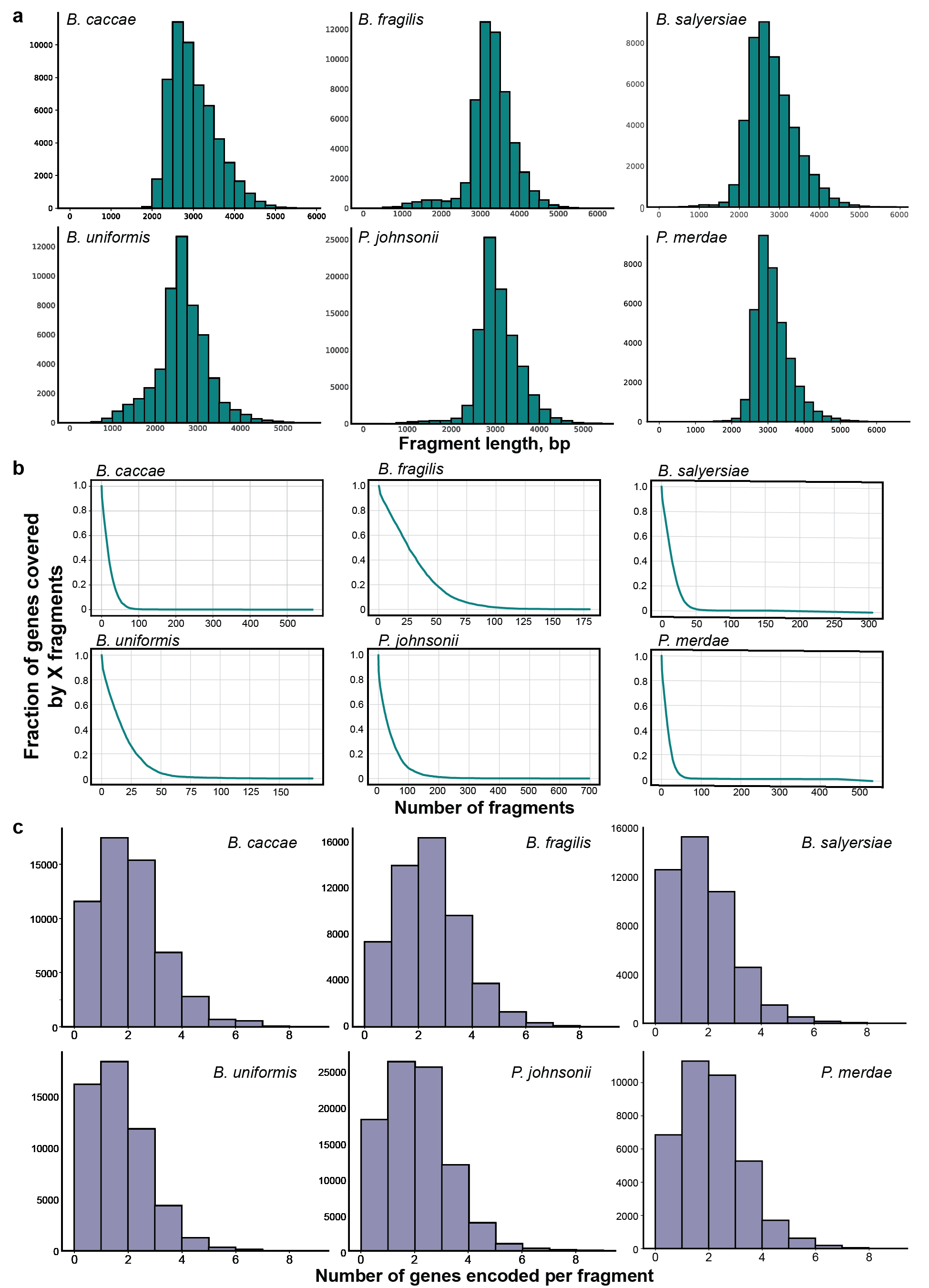


##### **Supplementary Figure 3.** Summary plots for six Boba-seq genomic libraries. Plots are generated as part of the mapping script. A) Histograms of fragment sizes. B) Cumulative gene coverage plots. C) Histograms of the number of full-length genes encoded per fragment with 0 as the first bin.


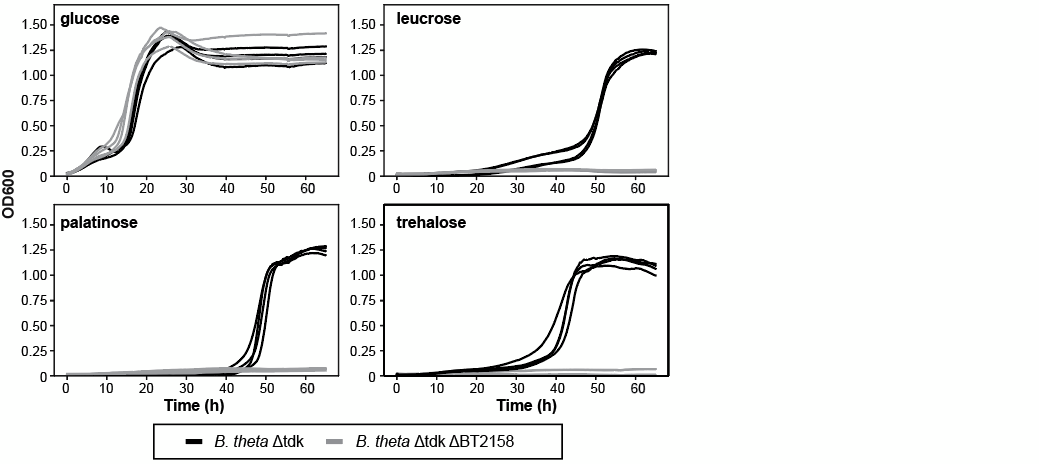


##### **Supplementary Figure 4.** Growth curves of *B. theta* Δ*tdk* parental strain and Δ*tdk*ΔBT2158 mutant on 20 mM glucose, leucrose, palatinose, or trehalose in the VB minimal medium. Each strain was grown in replicates of 4 per condition.


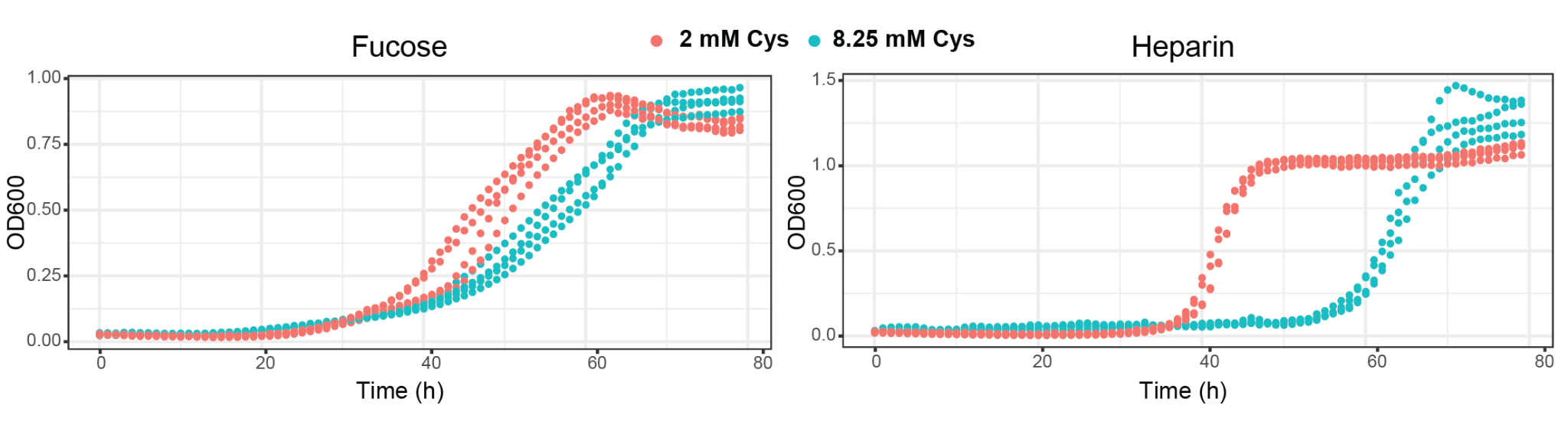


##### **Supplementary Figure 5.** Growth of wild-type *B. theta* is inhibited by the high cysteine concentration (8.25 mM) in the VB minimal medium. Examples are shown for 20 mM l-fucose and 10 mg/mL heparin in replicates of 4. A lower cysteine concentration (2 mM) resulted in improved growth.

##### **
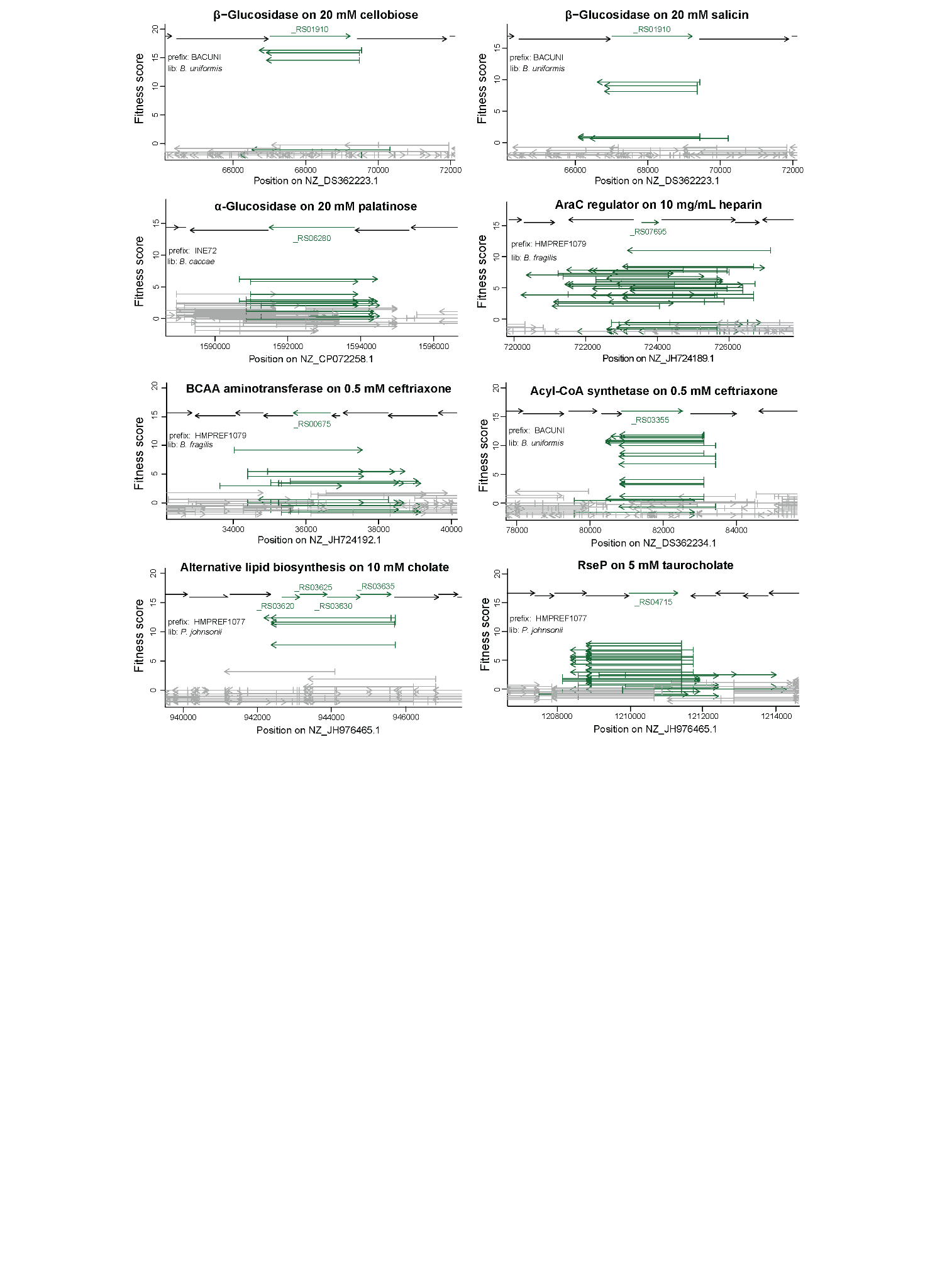
Supplementary Figure 6.** Fitness versus fragment plots of select gene hits across carbon utilization and stress assays. Each arrow represents a mapped fragment in the corresponding Boba-seq library. The beneficial gene(s) and all inserts covering the full-length gene(s) are highlighted in green. The genomic positions of the inserts within the source genome are displayed on the x-axis. The average fitness score of each fragment from two experiments are shown.

##### **
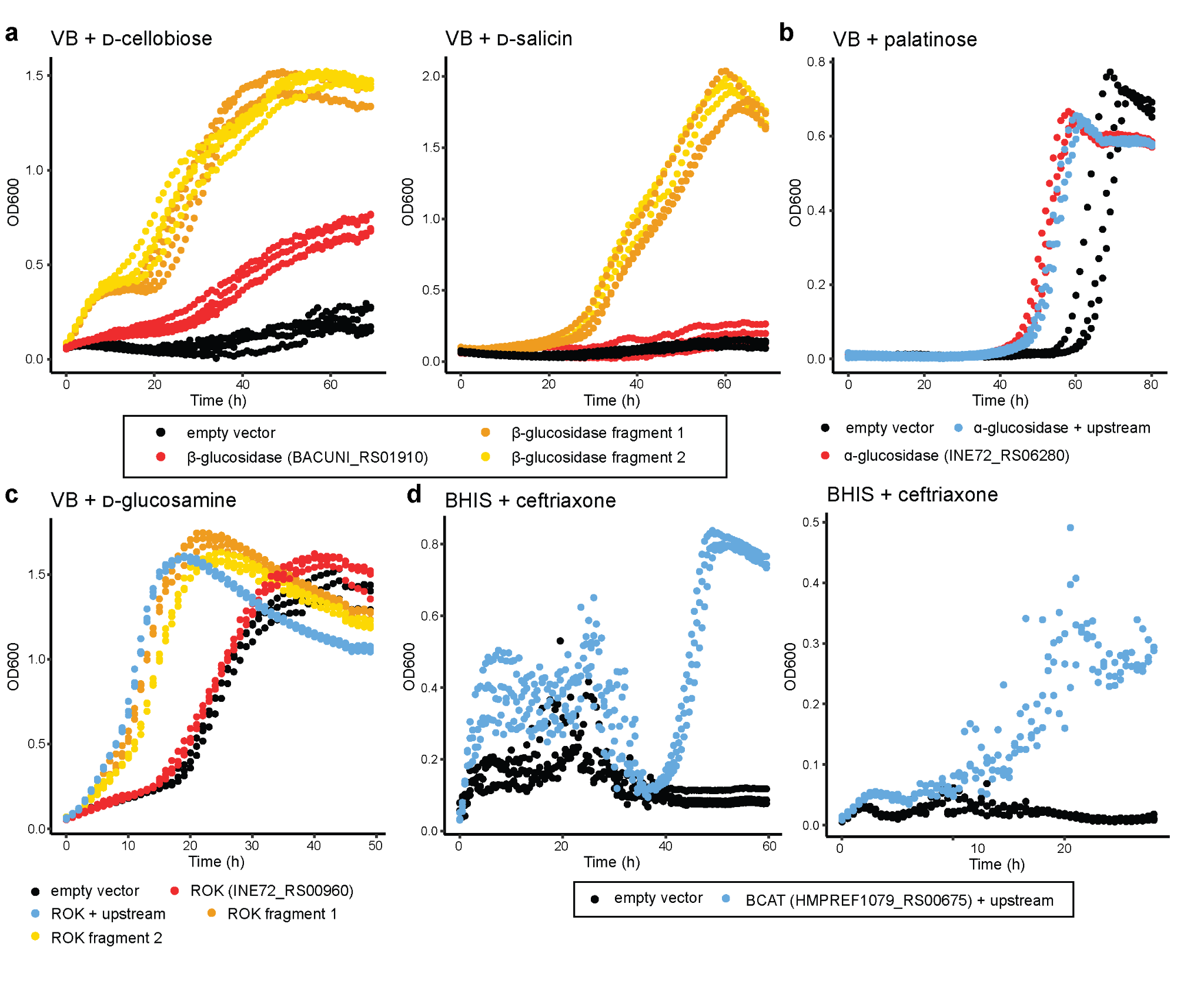
Supplementary Figure 7.** Growth benefits conferred by gene hits in individual *B. theta* strains. Each strain is conjugated with either a vector encoding the causative gene, the causative gene and the upstream 200 bp (or 100 bp for BCAT), a complete insert fragment from the library, or an empty vector. See Supplementary Data 7 for precise genomic boundaries, orientations, and fitness scores for each library fragment. Strains were constructed to confirm hits from A) d-cellobiose and d-salicin, B) palatinose, and C) d-glucosamine carbon substrates (20 mM) in the VB defined minimal medium. D) A branched-chain amino acid aminotransferase gene was confirmed to provide a benefit in BHIS with 1 mM ceftriaxone in two experiments. OD values are pathlength-corrected and blank-normalized. Each strain was grown in replicates of 3.

#####
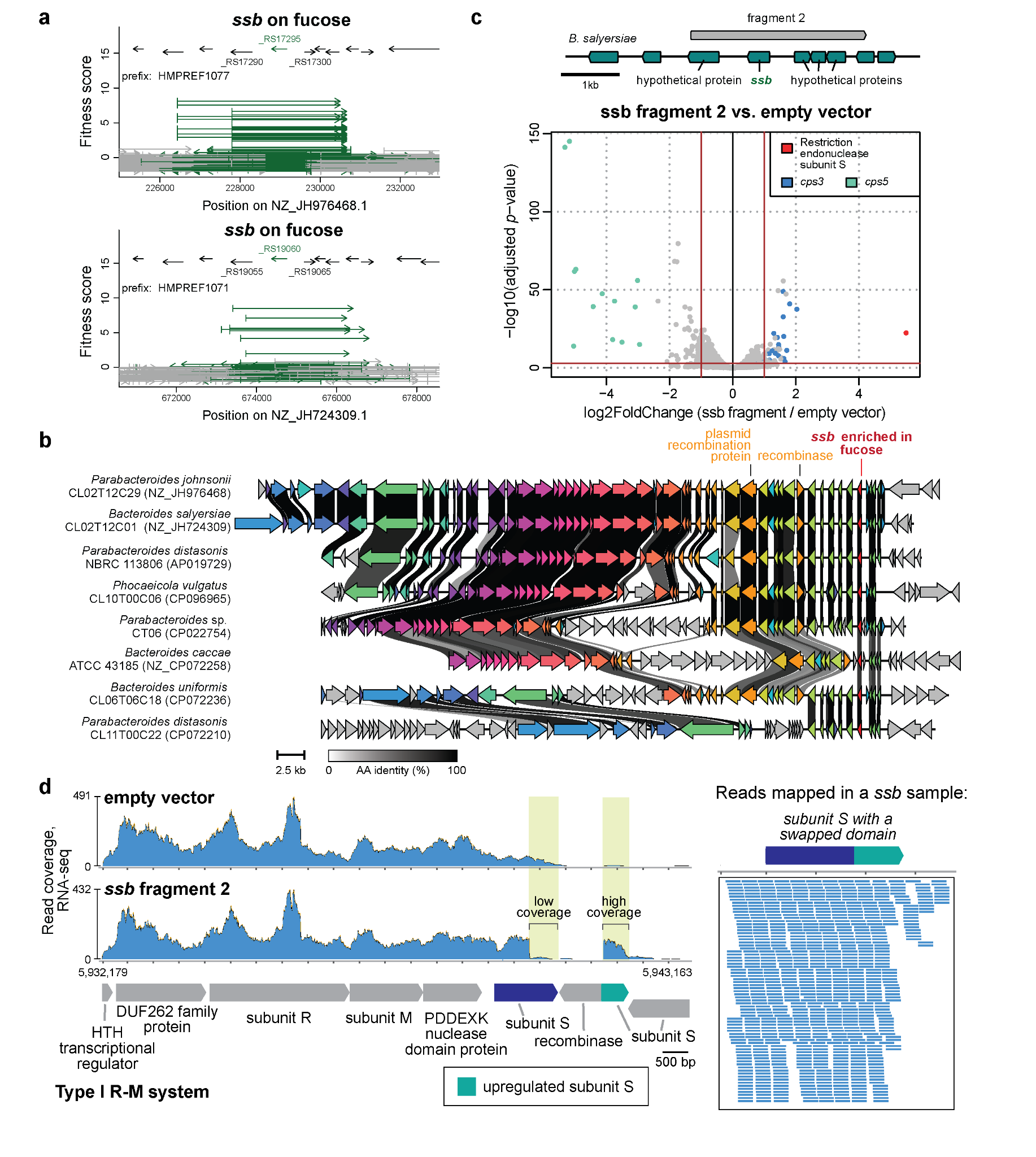


##### **Supplementary Figure 8.** A genomic region in two different isolates (*B. salyersiae* and *P. johnsonii*) encoding an SSB protein conferred a strong benefit for growth on 20 mM l-fucose. A) The fitness versus fragment plots for both regions. The fitness scores displayed are averages across 8 experiments, including assays with different reductants (Cys or DTT) and in the presence or absence of aTc. B) Gene neighborhoods of the *ssb* region among Bacteroidales suggest this region is part of a mobile genetic element inserted at different genomic locations. Insertions and rearrangements are observed downstream of the *ssb* gene along with a conserved plasmid recombination protein and a recombinase. Genomic comparisons were visualized using Clinker with an amino acid identity cutoff of 40%.^91^ C) Log2 ratios of fold-change and adjusted *p*-values (Wald test) from the RNA-seq experiment of *B. theta* encoding the *ssb* fragment 2 compared to an empty vector. Selected hits are labeled by color. D) Coverage plots of sense strand reads from RNA-seq data point to a genomic rearrangement that led to the upregulation of a subunit S (BT_RS22805/BT4522) part of a type I R-M system. Alignments were made using HISAT2 with default splice parameters on the strand-specific setting. Representative plots were generated using sense strand reads from empty_1 and ssb_1 samples. Sense strand reads from ssb_1 sample (*B. theta* encoding the *ssb* fragment 2) map across the subunit S gene formed after recombination.


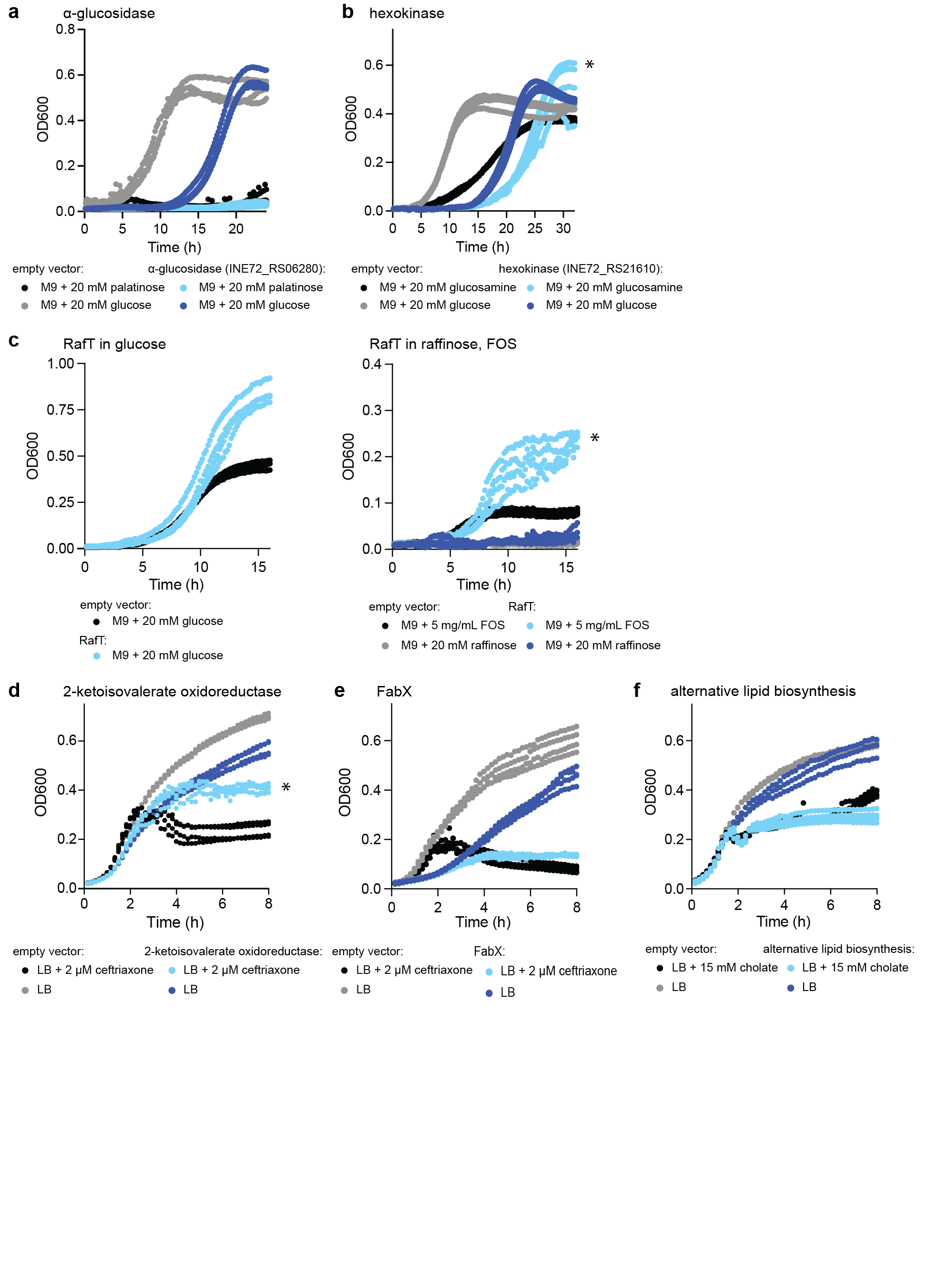


##### **Supplementary Figure 9.** Overexpression of beneficial gene hits in *E. coli* MG1655. Each strain was transformed with pTrcHis encoding the causative gene(s) verified to be beneficial for growth in *B. theta*. The empty pTrcHis vector is included for comparison. Growth in single carbon sources were performed for A) a-glucosidase (INE72_RS06280) in palatinose, B) hexokinase (INE72_RS21610) in d-glucosamine, C) RafT (HMPREF1079_RS07310) in raffinose and FOS. Glucose was included as a control for all. Growth in inhibitory compounds were formed for D) 2-ketoisovalerate oxidoreductase (HMPREF1077_RS19335, _RS17900, _RS17905, _RS17910) in 2 μM ceftriaxone, E) FabX (HMPREF1077_RS10180) in 2 μM ceftriaxone, and F) a gene cluster for alternative lipid biosynthesis (HMPREF1077_RS03620 to HMPREF1077_RS03635) in 15 mM cholate. Asterisks denote a growth benefit by the gene hit. Strains were grown in M9 defined minimal medium for carbon assays and LB for inhibitory compounds. Data from a representative experiment for each condition is shown. OD values are blank-normalized. Each strain was grown in replicates of 3 or 4 as displayed.

### **References**

90. Olm, M. R., Brown, C. T., Brooks, B. & Banfield, J. F. dRep: a tool for fast and accurate genomic comparisons that enables improved genome recovery from metagenomes through de-replication. *ISME J.* **11**, 2864–2868 (2017).

91. Gilchrist, C. L. M. & Chooi, Y.-H. clinker & clustermap.js: automatic generation of gene cluster comparison figures. *Bioinformatics* **37**, 2473–2475 (2021).
