# Supplementary material for: Barcoded overexpression screens in gut Bacteroidales identify genes with roles in carbon utilization and stress resistance
Source: Nat Commun. 2024 Aug 5;15:6618. doi: 10.1038/s41467-024-50124-3 (PMC11300592; doi:10.1038/s41467-024-50124-3)
Supplement: Supplementary file 3 — Description of Additional Supplementary Files [file 41467_2024_50124_MOESM3_ESM.docx]

File Name: Supplementary Data 1
Description: Strains, plasmids, oligonucleotides, and genetic parts used in this study.

File Name: Supplementary Data 2

Description: Summary of Boba-seq and RB-TnSeq libraries constructed in this study. Barcode diversity is reported for Boba-seq libraries in *E. coli* EC100D using BarSeq data. The percentage of barcodes mapped to a genomic region was computed using the PacBio and BarSeq data for Boba-seq libraries in *E. coli*.

File Name: Supplementary Data 3
Description: Conserved genes missing in genomic libraries in *E. coli*. This table excludes genes of over 1.5 kb or with nearly-identical duplicates in the same genome. Genes are only listed if a homolog (≥40% identity and ≥75% coverage, either from the same source genome or not) is also missing.

File Name: Supplementary Data 4
Description: Media and conditions used in fitness screens.

File Name: Supplementary Data 5
Description: Biologically consistent hits from all fitness assays performed in deletion mutants or in wild-type *B. theta*. Annotations of some hits are updated to reflect functional assignments. Hits for which Boba-seq data support a more precise functional annotation or association with a new phenotype are indicated; for this, hits without a homolog (not part of a protein cluster) are not considered, unless the gene(s) were verified to be benefitial in growth assays with individual strains. Set is indicated for the fitness assays in wild-type strain performed across multiple days. clusterBy lists the locus tags used for protein clustering. nHiBarcodes shows the total number of barcodes with a statistically significant fitness score (fitness ≥5 and z ≥4 in both replicates). avgHiFitness is the averaged fitness score (across replicates) for all barcodes that have a significant score.

File Name: Supplementary Data 6
Description: Gene hits with significant fitness scores and specific phenotypes in the *P. vulgatus* RB-TnSeq library.

File Name: Supplementary Data 7
Description: Strains isolated from complementation assays and library fragments cloned from Boba-seq libraries that were used in growth experiments.

File Name: Supplementary Data 8
Description: RNA-seq comparison of a strain expressing a ssb-containing fragment to an empty vector control. We show the top 100 upregulated and top 100 downregulated genes with adjusted p-value < 0.05 (Wald test, two-sided). For each gene and each sample, we show the normalized counts (from DEseq2's median of ratios).
