# Supplementary material for: Barcoded overexpression screens in gut Bacteroidales identify genes with roles in carbon utilization and stress resistance
Source: Nat Commun. 2024 Aug 5;15:6618. doi: 10.1038/s41467-024-50124-3 (PMC11300592; doi:10.1038/s41467-024-50124-3)
Supplement: Supplementary file 5 — Reporting Summary [file 41467_2024_50124_MOESM5_ESM.pdf]

nature portfolio

Corresponding author(s): Yolanda Huang, Adam Arkin

Last updated by author(s): 6/25/2024

Reporting Summary

Nature Portfolio wishes to improve the reproducibility of the work that we publish. This form provides structure for consistency and transparency in reporting. For further information on Nature Portfolio policies, see our Editorial Policies and the Editorial Policy Checklist.

Statistics

For all statistical analyses, confirm that the following items are present in the figure legend, table legend, main text, or Methods section.

n/a	Confirmed	
☐	☒	The exact sample size (*n*) for each experimental group/condition, given as a discrete number and unit of measurement
☐	☒	A statement on whether measurements were taken from distinct samples or whether the same sample was measured repeatedly
☐	☒	The statistical test(s) used AND whether they are one- or two-sided<br>*Only common tests should be described solely by name; describe more complex techniques in the Methods section.*
☒	☐	A description of all covariates tested
☐	☒	A description of any assumptions or corrections, such as tests of normality and adjustment for multiple comparisons
☐	☒	A full description of the statistical parameters including central tendency (e.g. means) or other basic estimates (e.g. regression coefficient) AND variation (e.g. standard deviation) or associated estimates of uncertainty (e.g. confidence intervals)
☐	☒	For null hypothesis testing, the test statistic (e.g. *F*, *t*, *r*) with confidence intervals, effect sizes, degrees of freedom and *P* value noted<br>*Give P values as exact values whenever suitable.*
☒	☐	For Bayesian analysis, information on the choice of priors and Markov chain Monte Carlo settings
☒	☐	For hierarchical and complex designs, identification of the appropriate level for tests and full reporting of outcomes
☒	☐	Estimates of effect sizes (e.g. Cohen's *d*, Pearson's *r*), indicating how they were calculated

Our web collection on statistics for biologists contains articles on many of the points above.

Software and code

Policy information about availability of computer code

Data collection	No specialized software was used for data collection. Standard Illumina base calling software were used to obtain sequencing reads.
Data analysis	Custom code used in this study is available on Github (https://github.com/OGalOz/Boba-seq; https://github.com/morgannprice/BobaseqFitness) with snapshots of all scripts used available on Figshare (https://doi.org/10.6084/m9.figshare.24195054). To generate Figure 2B, orgsToMarkers.pl script from the PaperBLAST code (https://github.com/morgannprice/PaperBLAST), HMMer3, a custom perl script (to generate a concatenated alignment), FastTree (2.1.11), and stat/reroot.pl (midpoint rooting) from the MicrobesOnline code (http://www.microbesonline.org/source/Genomics-18jun2009.tar.gz) were used. Lima (2.5.0) was used to demultiplex PacBio sequencing data. MultiCodes.pl (https://bitbucket.org/berkeleylab/feba) was used to demultiplex and analyze barcode sequencing data. HISAT2 (2.2.1), featureCounts (part of Subread 2.0.1 package), and DESeq2 (1.38.2) were used to analyze RNA-seq data. For RB-TnSeq data, BarSeqR.pl from the feba code base (https://bitbucket.org/berkeleylab/feba) was used to compute gene fitness values and t-like test statistics.

For manuscripts utilizing custom algorithms or software that are central to the research but not yet described in published literature, software must be made available to editors and reviewers. We strongly encourage code deposition in a community repository (e.g. GitHub). See the Nature Portfolio guidelines for submitting code & software for further information.

Data

Policy information about availability of data

All manuscripts must include a data availability statement. This statement should provide the following information, where applicable:

- Accession codes, unique identifiers, or web links for publicly available datasets
- A description of any restrictions on data availability
- For clinical datasets or third party data, please ensure that the statement adheres to our policy

Boba-Seq and RNA-seq data generated in this study is archived at https://doi.org/10.6084/m9.figshare.24195054. This also includes the plasmid sequences, genomes and annotations used for library mapping, results from the mapping step of Boba-seq libraries, a table of all samples analyzed in fitness screens, the R image of fitness analyses, all-versus-all protein similarity results, and RNA-seq data. Figure 3D is generated using publicly available RB-TnSeq data from https://fit.genomics.lbl.gov/cgi-bin/singleFit.cgi?orgId=Btheta&locusId=351608&showAll=0. RB-TnSeq data generated in this study is archived at https://doi.org/10.6084/m9.figshare.25620471 and can be viewed in the Fitness Browser (http://fit.genomics.lbl.gov).
There are no restrictions on the availability of data generated in this study.

Research involving human participants, their data, or biological material

Policy information about studies with human participants or human data. See also policy information about sex, gender (identity/presentation), and sexual orientation and race, ethnicity and racism.

Reporting on sex and gender	Not applicable.
Reporting on race, ethnicity, or other socially relevant groupings	Not applicable.
Population characteristics	Not applicable.
Recruitment	Not applicable.
Ethics oversight	Not applicable.

Note that full information on the approval of the study protocol must also be provided in the manuscript.

Field-specific reporting

Please select the one below that is the best fit for your research. If you are not sure, read the appropriate sections before making your selection.

- [x] Life sciences
- [ ] Behavioural & social sciences
- [ ] Ecological, evolutionary & environmental sciences

For a reference copy of the document with all sections, see nature.com/documents/nr-reporting-summary-flat.pdf

Life sciences study design

All studies must disclose on these points even when the disclosure is negative.

Sample size	No formal sample size calculations were performed to predetermine sample size. For each fitness assay of Boba-seq and RB-TnSeq libraries, we used two biological replicates (grown separately but inoculated on the same day). Two replicates is sufficient because we were only interested in large effects and because replicates usually gave consistent results (as described in the Results). For growth curves of individual strains, we used 2-4 replicates because we were interested in large effects. For the RNA-seq experiment, we included three samples in each group to identify strong effects on transcript levels.
Data exclusions	There were two samples with insufficient reads, set4IT038 (Buni lib in delBT2158, trehalose) and fit2S121 (0.05 mM levofloxacin, wt host). Due to the lack of a replicate, we don't report results for either of these conditions.
Replication	All fitness assays of Boba-seq and RB-TnSeq libraries were carried out in at least two replicates on the same day. All growth experiments of individual strains were performed with replicates of 2-4 for each condition. Data from growth experiments performed on different days (not shown) were also obtained for each hit and led to similar results as the representative experiment shown. Three cultures of each strain were analyzed in the RNA-seq experiment. This is all described in the Methods section.
Randomization	There was no randomization of samples in this study because it is not relevant to this study.
Blinding	There was no blinding of samples in this study because it is not relevant to this study.

Reporting for specific materials, systems and methods

We require information from authors about some types of materials, experimental systems and methods used in many studies. Here, indicate whether each material, system or method listed is relevant to your study. If you are not sure if a list item applies to your research, read the appropriate section before selecting a response.

Materials & experimental systems

n/a	Involved in the study
☒	☐ Antibodies
☒	☐ Eukaryotic cell lines
☒	☐ Palaeontology and archaeology
☒	☐ Animals and other organisms
☒	☐ Clinical data
☒	☐ Dual use research of concern
☒	☐ Plants

Methods

n/a	Involved in the study
☒	☐ ChIP-seq
☒	☐ Flow cytometry
☒	☐ MRI-based neuroimaging

Plants

Seed stocks	*Report on the source of all seed stocks or other plant material used. If applicable, state the seed stock centre and catalogue number. If plant specimens were collected from the field, describe the collection location, date and sampling procedures.*
Novel plant genotypes	*Describe the methods by which all novel plant genotypes were produced. This includes those generated by transgenic approaches, gene editing, chemical/radiation-based mutagenesis and hybridization. For transgenic lines, describe the transformation method, the number of independent lines analyzed and the generation upon which experiments were performed. For gene-edited lines, describe the editor used, the endogenous sequence targeted for editing, the targeting guide RNA sequence (if applicable) and how the editor was applied.*
Authentication	*Describe any authentication procedures for each seed stock used or novel genotype generated. Describe any experiments used to assess the effect of a mutation and, where applicable, how potential secondary effects (e.g. second site T-DNA insertions, mosiacism, off-target gene editing) were examined.*
